# Single-cell transcriptomic profiling of human pancreatic islets reveals genes responsive to glucose exposure over 24 h

**DOI:** 10.1007/s00125-024-06214-4

**Published:** 2024-07-05

**Authors:** Caleb M. Grenko, Henry J. Taylor, Lori L. Bonnycastle, Dongxiang Xue, Brian N. Lee, Zoe Weiss, Tingfen Yan, Amy J. Swift, Erin C. Mansell, Angela Lee, Catherine C. Robertson, Narisu Narisu, Michael R. Erdos, Shuibing Chen, Francis S. Collins, D. Leland Taylor

**Affiliations:** 1 Center for Precision Health Research, National Human Genome Research Institute, National Institutes of Health, Bethesda, MD USA; 2 Graduate School of Biomedical Sciences, Mayo Clinic, Rochester, MN USA; 3 British Heart Foundation Cardiovascular Epidemiology Unit, Department of Public Health and Primary Care, University of Cambridge, Cambridge, UK; 4 Heart and Lung Research Institute, University of Cambridge, Cambridge, UK; 5 Department of Surgery, Weill Cornell Medicine, New York, NY USA; 6 Center for Genomic Health, Weill Cornell Medicine, New York, NY USA; 7 Department of Computational Medicine and Bioinformatics, University of Michigan, Ann Arbor, MI USA

**Keywords:** Genetics, Genomics, GSIS, Islets, Single-cell, Transcriptomics, Type 2 diabetes

## Abstract

**Aims/hypothesis:**

Disruption of pancreatic islet function and glucose homeostasis can lead to the development of sustained hyperglycaemia, beta cell glucotoxicity and subsequently type 2 diabetes. In this study, we explored the effects of in vitro hyperglycaemic conditions on human pancreatic islet gene expression across 24 h in six pancreatic cell types: alpha; beta; gamma; delta; ductal; and acinar. We hypothesised that genes associated with hyperglycaemic conditions may be relevant to the onset and progression of diabetes.

**Methods:**

We exposed human pancreatic islets from two donors to low (2.8 mmol/l) and high (15.0 mmol/l) glucose concentrations over 24 h in vitro. To assess the transcriptome, we performed single-cell RNA-seq (scRNA-seq) at seven time points. We modelled time as both a discrete and continuous variable to determine momentary and longitudinal changes in transcription associated with islet time in culture or glucose exposure. Additionally, we integrated genomic features and genetic summary statistics to nominate candidate effector genes. For three of these genes, we functionally characterised the effect on insulin production and secretion using CRISPR interference to knock down gene expression in EndoC-βH1 cells, followed by a glucose-stimulated insulin secretion assay.

**Results:**

In the discrete time models, we identified 1344 genes associated with time and 668 genes associated with glucose exposure across all cell types and time points. In the continuous time models, we identified 1311 genes associated with time, 345 genes associated with glucose exposure and 418 genes associated with interaction effects between time and glucose across all cell types. By integrating these expression profiles with summary statistics from genetic association studies, we identified 2449 candidate effector genes for type 2 diabetes, HbA_1c_, random blood glucose and fasting blood glucose. Of these candidate effector genes, we showed that three (*ERO1B*, *HNRNPA2B1* and *RHOBTB3*) exhibited an effect on glucose-stimulated insulin production and secretion in EndoC-βH1 cells.

**Conclusions/interpretation:**

The findings of our study provide an in-depth characterisation of the 24 h transcriptomic response of human pancreatic islets to glucose exposure at a single-cell resolution. By integrating differentially expressed genes with genetic signals for type 2 diabetes and glucose-related traits, we provide insights into the molecular mechanisms underlying glucose homeostasis. Finally, we provide functional evidence to support the role of three candidate effector genes in insulin secretion and production.

**Data availability:**

The scRNA-seq data from the 24 h glucose exposure experiment performed in this study are available in the database of Genotypes and Phenotypes (dbGap; https://www.ncbi.nlm.nih.gov/gap/) with accession no. phs001188.v3.p1. Study metadata and summary statistics for the differential expression, gene set enrichment and candidate effector gene prediction analyses are available in the Zenodo data repository (https://zenodo.org/) under accession number 11123248. The code used in this study is publicly available at https://github.com/CollinsLabBioComp/publication-islet_glucose_timecourse.

**Graphical Abstract:**

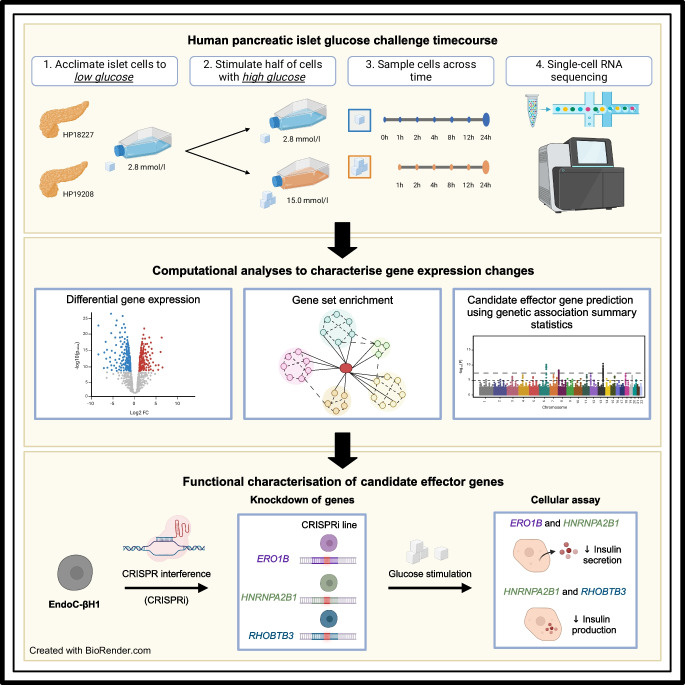

**Supplementary Information:**

The online version contains peer-reviewed but unedited supplementary material available at 10.1007/s00125-024-06214-4.


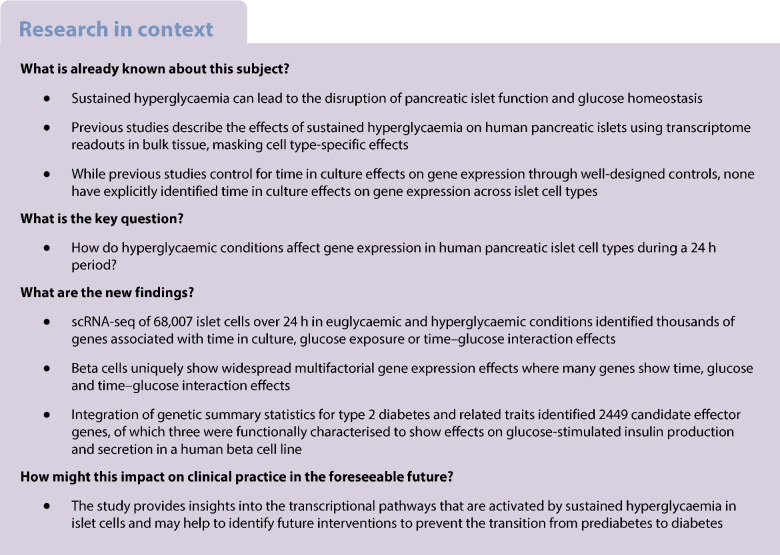


## Introduction

Type 2 diabetes and related complications are among the leading causes of death globally [1]. Clinical and genetic studies highlight the central role of pancreatic islet dysfunction and disrupted glucose homeostasis in the development of sustained hyperglycaemia and type 2 diabetes (reviewed in [2, 3]). Within the pancreatic islet, multiple cell types have been implicated in type 2 diabetes progression, most notably beta cells which secrete insulin in response to glucose stimulation [4], but also other cell types including alpha cells [5] and delta cells [6]. Common variant genetic association studies have identified >500 genetic signals associated with type 2 diabetes and type 2 diabetes-related traits [7], promising to deliver clues to the genes and molecular pathways underlying type 2 diabetes development and progression. However, most genetic signals identified to date lie outside protein-coding genes, masking the ‘effector genes’ responsible for driving the genetic association.

One approach to help identify and understand the genes that contribute to type 2 diabetes pathogenesis and progression is to explore the effects of physiologically relevant conditions, such as hyperglycaemia, on human islet gene expression. To date, human islet glucose-stimulus studies have shown the effects of hyperglycaemia on genes related to insulin secretion [8] and oxidative stress [9, 10]. The molecular picture from these studies is consistent with our understanding of type 2 diabetes pathophysiology; under normal conditions, transient increases in blood glucose stimulate insulin secretion. However, under the sustained hyperglycaemic conditions that occur in type 2 diabetes, the continual demands of insulin production lead to glucotoxicity and apoptosis of beta cells [11] (and possibly other islet cell types [12]), further exacerbating the type 2 diabetes condition. Despite the known importance of multiple islet cell types in the context of diabetes [4–6], to date, transcriptomic studies examining the effects of hyperglycaemia on primary human islets have used gene expression readouts from bulk islet tissue [8–10], thus masking cell-type-specific expression signatures that may be relevant to diabetes.

In this study, we characterise the transcriptional changes associated with sustained glucose exposure across islet cell types by exposing human pancreatic islets from two donors to euglycaemic (2.8 mmol/l) and hyperglycaemic (15.0 mmol/l) conditions in vitro and performing single-cell RNA-seq (scRNA-seq) at seven time points over 24 h. The results from this study provide a high-resolution view of the effects of euglycaemic and hyperglycaemic conditions on islet cell types through time and should help guide future experiments to understand the molecular mechanisms that lead to islet dysfunction in disease states like type 2 diabetes.

## Methods

### Ethics statement

The pancreatic islets used in this study were isolated from cadaverous donors whose organs were consented for research. As per the United States’ Office for the Protection of Research Subjects policy, islets obtained from non-living individuals do not fall under the guidelines of human subject research. All experimental protocols performed for this study were approved under National Institutes of Health (NIH) guidelines.

### Islet procurement and processing

We obtained purified human pancreatic islets from two individuals through Prodo Laboratories (Aliso Viejo, CA, USA; electronic supplementary material [ESM]  Table 1; ESM Human Islet Checklist). After receiving the islets, we equilibrated them to 37°C for 1 h in 2.8 mmol/l glucose media. Prior to shipment, islets were characterised by Prodo Laboratories using a glucose-stimulated insulin release assay (ESM Fig. 1). See ESM Methods for details.

### Genotyping

We genotyped pancreatic islets from both donors as previously described [13]. We imputed filtered genotypes using Minimac4 v1.7.3 [14] on the TOPMed Imputation Server [15] and removed SNPs with an imputation *r*^2^≤0.3. See ESM Methods for details.

### Single-cell RNA sequencing of glucose-stimulated pancreatic islets

We exposed pancreatic islets to either low (2.8 mmol/l) or high (15 mmol/l) glucose for 24 h and performed single-cell RNA-seq (scRNA-seq) at a baseline 2.8 mmol/l glucose before starting the experiment (time point 0) and at 1, 2, 4, 8, 12 and 24 h time points. We incubated aliquots of 2000 islet equivalents (IEQs) at 37°C for the duration of the experiment and sampled wells at each time point. We performed experiments in duplicate, resulting in two replicates for each donor, time point and glucose condition. We dissociated the islets and performed scRNA-seq using the 10X Genomics Chromium platform (10X Genomics, Pleasanton, CA, USA) according to the manufacturer’s instructions. See ESM Methods for details.

### Single-cell RNA-seq processing and quality control

We used CellRanger v3.1.0 (10X Genomics, Pleasanton, CA, USA) to process and align reads to GRCh38.p13, identify cell-containing droplets and generate cell × gene count matrices. We used a two-step approach with DecontX [16] to achieve the following objectives: (1) remove droplets with a high ambient transcript contamination from the single-cell sequencing experiment; and (2) adjust the raw counts matrix for the ambient expression signature. For the first pass, we applied DecontX to count matrices from CellRanger and cell type clusters derived from Seurat v4.3.0 [17] and removed cell-containing droplets with >10% ambient contamination from the CellRanger gene count matrix. For the second pass, we re-ran the DecontX workflow (including Seurat clustering) with the filtered gene count matrix, removed droplets with >10% ambient contamination, and used the DecontX-adjusted count matrix.

To retain high-quality cells, we filtered multiplets using scrublet v0.2.1 [18], cells with >50% unique molecular identifier (UMI) counts from the mitochondrial genome, outlier cells based on the total number of UMIs and then on the number of genes expressed (≥1 count), and contaminated cells (identified by comparing sequencing reads with donor genotypes). We used scanpy v1.6.0 [19] to filter lowly expressed (≥1 count in ≤5 cells), mitochondrial and ribosomal genes and normalised UMI counts to the log-transformed counts per 10,000 (log_*e*_[CP10K+1]). See ESM Methods for details.

### Cell type annotation

We mean-centred and scaled the cell × gene expression matrix of the 2000 most variable genes across samples and performed principal component (PC) analysis using scanpy v1.6.0 [19]. We used a scree plot [20] to select nine PCs for downstream analyses and used harmony v0.0.5 [21] to adjust PCs for sample-specific batch effects. With the harmony-adjusted PCs, we clustered the cells using an iterative parameter sweep of a Leiden graph-based algorithm v0.8.3 [22]. To optimise the clustering, we performed a parameter sweep and trained/tested a neural-network-based cell type classifier (keras v2.4.3; https://keras.io/) for each configuration. We evaluated parameter configurations using Matthew’s correlation coefficient, selected a cluster resolution of 0.25 for the final analysis, and identified eight clusters. To determine the cell type identity of clusters, we used well-established marker genes for islet cell types. Finally, we applied the cell type classifier to cells that were previously filtered due to mitochondrial count thresholds and recovered cells with a cell type predictor >0.5. See ESM Methods for details.

### Time interpolation

Within each cell type, donor and glucose condition, we calculated the interpolated time as the weighted sum of the sampled time from each cell and the 75 nearest neighbours identified using scvelo v0.2.4 [23]. See ESM Methods for details.

### Differential gene expression analysis

We performed differential gene expression (DGE) analysis using MAST v1.20.0 [24], including cell complexity (i.e. the number of genes detected per cell) as a fixed effect covariate, participant and experiment identifiers as random effect covariates, and additional, model-specific fixed effect covariates (see ESM Methods).

For the discrete time DGE models, we fit separate models for each cell type and time point: (1) ‘basal vs high’ (BvH; comparing basal and high glucose cells across time); (2) ‘basal vs low’ (BvL; comparing basal and low glucose cells across time); and (3) ‘low vs high’ (LvH; comparing low and high glucose cells across time). For the continuous time DGE models, we fit separate models for each cell type: (1) ‘continuous time’ (to test for time effects); (2) ‘glucose’ (to test for glucose effects); and (3) ‘time–glucose interaction’ (to test for an interaction effect between time and glucose concentration).

For each model, we controlled for the false discovery rate (FDR) using the Benjamini–Hochberg (BH) procedure [25]. We removed genes with median counts per 10,000 (CP10K)<1 prior to fitting models for each cell type. See ESM Methods for details.

### Gene ontology enrichment and clustering analysis

For each DGE model (e.g. BvL at 1 h, BvL at 2 h, continuous time, glucose), we identified enriched gene ontology (GO) terms from the ‘biological process’ ontology using clusterProfiler v4.8.3 [26]. We controlled for the number of tests performed using the BH procedure [25]. To visualise results, we modelled the semantic similarity of enriched GO terms (FDR<5%) using GOSemSim v2.26.1 [27] and created network plots using enrichplot v1.20.3 [28]. See ESM Methods for details.

### Nomination of candidate effector genes for type 2 diabetes and type 2 diabetes-related traits

We nominated candidate effector genes using the polygenic priority score (PoPS) method v0.2 [29] and publicly available summary statistics for type 2 diabetes, fasting blood glucose, random blood glucose and HbA_1c_. For the genomic feature matrices, we constructed a ‘control’ matrix of housekeeping genes and a ‘test’ matrix comprising features from the single-cell data presented in this study (as performed by Weeks et al [29]), with the addition of cell type gene expression specificity values and test statistics from the differential expression models (excluding the BvH model). We calculated empirical *p* values from a null distribution (obtained by permuting ‘test’ matrix and repeating analysis 1000 times) and controlled the FDR across all genes considered using the BH procedure [25]. We compared the candidate effector genes with the 132 effector genes from the Type 2 Diabetes Knowledge Portal (https://t2d.hugeamp.org). See ESM Methods for details.

### CRISPR interference experiments

We designed and synthesised three different guide RNAs (gRNAs) targeting transcription start site regions of each candidate gene as well as two non-targeting gRNAs (ESM Table 2). As previously described [30], we cloned the gRNAs into the CRISPR interference (CRISPRi) vector and transfected HEK293T cells (ATCC, Manassas, VA, USA) with the lentivirus packaging plasmids and the CRISPRi plasmids. After concentrating and resuspending the vial suspension, we seeded around 1 million EndoC-βH1 [31] cells in six-well plates and infected them with 0.2 ml of viral suspension (supplemented with polybrene). At 72 h post-transduction, we exposed cells to 2 µg/ml puromycin to select for the infected cells. See ESM Methods for details.

### Quantitative RT-PCR experiments

We performed quantitative RT-PCR (qRT-PCR) as previously described [32] using primers specific to each candidate gene and the reference gene (*GAPDH*; ESM Table 3). See ESM Methods for full details.

### Glucose stimulation experiment in CRISPRi EndoC-βH1 cells

We seeded 1 million CRISPRi cells and the control cells in 12-well plates, fasted the cells in 1 ml low glucose KRBH at 37°C for 1 h, and exposed cells to low (2.8 mmol/l) and high (15 mmol/l) glucose KRBH at 37°C for 24 h. At 1 h, we measured extracellular insulin content in the supernatant fraction. At 24 h, we measured intracellular insulin content after removing residual medium and lysing the cells. At both time points, we calculated the insulin stimulation index as the ratio of the mean insulin content between the high and low glucose conditions. We calculated the 95% CI of the high–low means using Fieller’s method [33, 34] and compared the gene-targeting CRISPRi experiments with the control experiments using Welch’s *t* test [35]. See ESM Methods for details.

### Comparison with results of previous bulk islet transcriptome studies

We compared the glucose-associated genes identified in this study with results from previous transcriptomic studies of glucose stimulation in bulk islets [8–10] (see ESM Methods for details).

## Results

### Single-cell RNA sequencing of glucose-stimulated human pancreatic islets

**Fig. 1:**
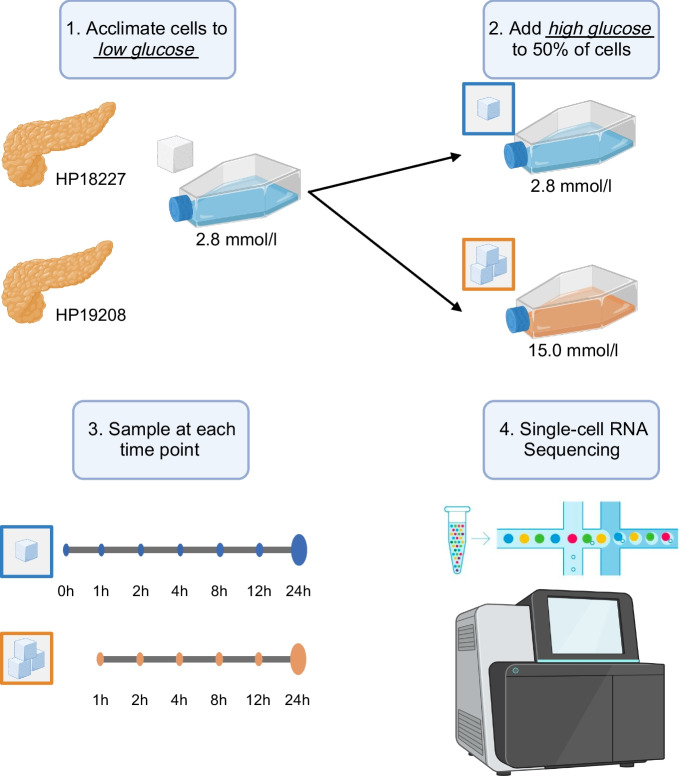
Graphical overview of this study. (1) Upon receipt, we acclimated pancreatic islets from two donors (donor IDs indicated) to low glucose conditions (2.8 mmol/l) for 1 h. (2) After acclimation, we exposed half of the islets to high glucose (15.0 mmol/l) and kept the other half in low glucose conditions (2.8 mmol/l). (3) At 0, 1, 2, 4, 8, 12 and 24 h time points, we sampled islets and (4) performed single-cell RNA sequencing. Time point 0 corresponds to cells after 1 h low-glucose acclimation, prior to starting the stimulation experiment. Created with BioRender.com

We obtained human pancreatic islets from two donors and acclimated the islets to a basal, euglycaemic media of 2.8 mmol/l glucose (50 mg/dl) over 1 h (Fig. 1). After 1 h, we sampled a subset of cells and performed scRNA-seq. For the remaining cells, we either maintained them in the low (basal) glucose condition or exposed them to a high (hyperglycaemic) glucose concentration of 15.0 mmol/l (270 mg/dl). We subsequently sampled cells at six additional time points for scRNA-seq over the course of 24 h (cells remained exposed to glucose over the entire time course). We refer to cells from islets sampled after 1 h of acclimation in 2.8 mmol/l glucose as ‘basal’ cells (time point 0 h) and cells from islets sampled at later time points as either ‘low’ or ‘high’ glucose cells (time point 1–24 h). After quality control procedures (see Methods), we obtained 68,007 cells spanning eight cell types, including endocrine cells (beta 29.0%, alpha 25.4%, delta 4.5% and gamma 2.8%), exocrine cells (acinar 26.1% and ductal 11.7%), macrophages (0.3%) and endothelial cells (0.1%; ESM Figs 2–4). For subsequent analysis, we removed macrophage and endothelial cells, as these cells were rare and poorly represented across donors, time points and glucose conditions (<80 cells at each time point; ESM Fig. 4).

### Time-point-specific effects of glucose induction

We fit three discrete models to characterise the transcriptomic response of islet cell types to glucose stimulation at each time point. First, in the BvH glucose model, we compared the gene expression of cells in the basal state (2.8 mmol/l glucose, 0 h time point) with cells in high glucose at each time point. This model identifies transcriptomic effects of high glucose at various time points but confounds the impact of high glucose with time in culture. Second, in the BvL glucose model, we compared the expression of cells in the basal state (2.8 mmol/l glucose, 0 h time point) with cells that remained in the low glucose condition at every other time point, thus isolating time in culture effects while removing the glucose concentration as a confounding factor. Finally, we fit a third model within each time point, comparing cells exposed to low glucose against cells exposed to high glucose (LvH) to identify glucose-related effects while controlling for time.

**Fig. 2:**
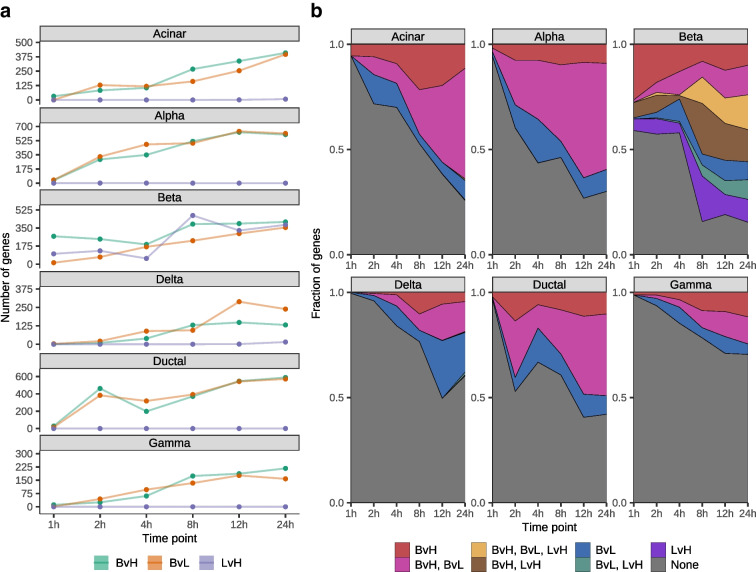
Differentially expressed genes in discrete time models. (**a**) Number of associated genes (FDR<5%) at each time point for three models with time as a discrete variable: BvL (orange); BvH (green); and LvH (purple). (**b**) Fraction of associated genes that are model-specific or shared across models at each time point. Colour denotes model or combination of models. ‘None’ indicates the fraction of genes without an association in any model

For the BvH and BvL models, the number of associated genes increased over time across all cell types (Fig. 2a). By contrast, for the LvH model (which best isolates glucose-related effects), we observed very few transcriptional changes, except in the beta cells where the total number of associated genes peaked at 8 h (Fig. 2a). For cell types other than beta cells, the proportion of genes differentially expressed in both the BvH and BvL models increased across time, suggesting that time in culture may confound the BvH results, as anticipated (Fig. 2b). Indeed, when we compared the signed −log_10_(*p* values) across models, we found that the BvH and BvL values but not the LvH values were strongly correlated (ESM Fig. 5). Combined, these results confirm the following: (1) that the BvH model confounds time and glucose effects; and (2) that the BvL and LvH models best isolate time and glucose effects, respectively. Therefore, we chose to focus on the BvL and LvH models for subsequent analyses (ESM Fig. 6).

To further characterise the transcriptional response to time in culture and glucose exposure, we calculated the overlap of associated genes (FDR<5%) across time points within each model and cell type (ESM Figs 7, 8) and determined the earliest time point that a gene showed an association (ESM Fig. 9). Focusing on the BvL model (ESM Figs 7, 9a), we observed a similar pattern across all cell types: a sustained cascade of transcription throughout the 24 h. Turning to the LvH model (ESM Fig. 8), the time point with the greatest number of uniquely associated genes varied across cell types: 24 h for acinar and delta cells, both 2 h and 4 h for alpha cells (where we found one associated gene) and 8 h for beta cells. Interestingly, for beta cells, we found that the number of differentially expressed genes increased drastically at 8 h and was sustained up to 24 h, indicating that a robust transcriptomic response of beta cells to continuous glucose exposure requires approximately 8 h. The timing of the transcriptomic response of beta cells to glucose exposure was also apparent when we considered the earliest time at which a differentially expressed gene was identified; in beta cells, roughly 65.6% (433/660) of all genes differentially expressed in the LvH model showed expression differences on or after 8 h (ESM Fig. 9b). This observation was unique to beta cells.

To describe the biological processes underlying the transcriptional changes, we identified enriched gene sets in the differentially expressed genes using the ‘biological process’ GO database and then clustered the enriched GO terms (FDR<5%) based on their semantic similarity (ESM Figs 10, 11). For the BvL model, we identified many GO terms related to stress response and cellular signalling across all cell types (ESM Fig. 10). Terms associated with earlier time points (e.g. 2 h and 4 h) involved translational activation and cellular response to stimuli, and those associated with later time points (e.g. 8 h, 12 h and 24 h) involved cellular respiration processes and transcriptional activation. For the LvH model, we found many terms enriched in beta cells, most of which were associated with metabolism, cellular respiration, cellular signalling, protein folding and protein localisation, consistent with known mechanisms of beta cell response to glucose stimulation (reviewed in [36, 37]).

### Temporal dynamics of gene expression in response to glucose induction

While discrete models can effectively identify time-specific effects (ESM Figs 5–11), such models fail to make use of the full potential of the data as they do not simultaneously model gene expression across all time points, leading to reduced power to detect effects common to multiple time points and an inability to model more complex relationships such as interaction effects between time and glucose concentration (time–glucose effects). Treating time as a continuous variable, we fit a series of models to identify gene expression patterns associated with time (with glucose as a covariate), glucose concentration (with time as a covariate) and time–glucose (with time and glucose as covariates).

To model time, we considered two metrics: (1) sampled time (i.e. the experimental time point); and (2) interpolated time (ESM Figs 12, 13), a metric that models similarities between cells based on the assumption that cells sampled at various time points are not synchronised at the exact same response phase (i.e. some cells sampled at 8 h may have lagged in their response to stimulation and therefore have an expression profile more similar to 6 h than 8 h). We fit the time, glucose and time–glucose models using the two different time metrics and found a slight boost in power using interpolated time over sampled time with concordant directions of effect (ESM Fig. 13b, c), suggesting that interpolated time represents the phase of cellular response more accurately than sampled time. Therefore, in all subsequent continuous models, we used interpolated time.

Across the three continuous models, we identified 1321 genes with expression patterns associated with time, glucose and time–glucose (FDR<5%; ESM Figs 13c, 14). As anticipated, compared with the discrete models, the continuous models identified many effects that were not detected previously (ESM Fig. 15).

**Fig. 3:**
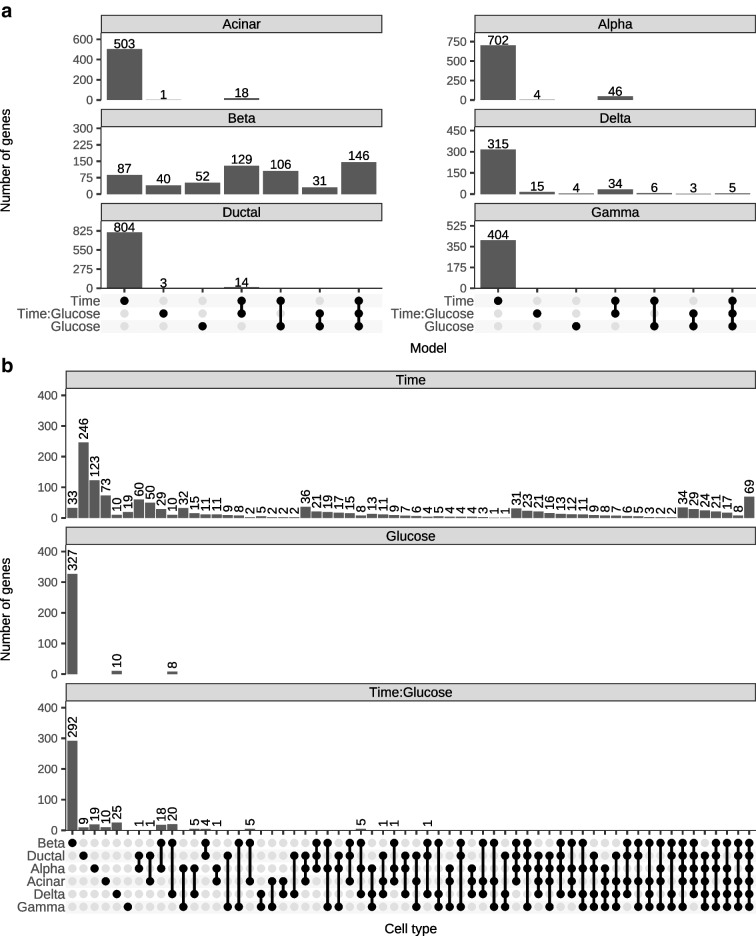
Differentially expressed genes in continuous time models. (**a**) Number of associated genes (FDR<5%) in each cell type that are model-specific or shared across models. (**b**) Number of associated genes (FDR<5%) from each model that are cell type-specific or shared across cell types

We calculated the overlap of associated genes between models within cell types (Fig. 3a) and between cell types within models (Fig. 3b). We found that across the three models within a given cell type, very few genes exhibited exclusive glucose effects (Fig. 3a). Beta cells had the most genes with glucose-specific effects, with 52 genes including those with known roles in beta cell function such as *CDC42* [38] and *FIS1* [39]. By contrast, many genes were associated exclusively with time in culture, with the association being notably strong in alpha, delta, acinar, gamma and ductal cells but absent in beta cells. Compared with all cell types, beta cells had the most genes with time–glucose interaction effects, featuring several well-established type 2 diabetes genes, including *INS* [40], *ABCC8* [41, 42], *SLC30A8* [43, 44] and *PCBD1* [45]. When we considered the overlap of genes across cell types within each model (Fig. 3b), we found that the genes associated with time in culture showed dispersed patterns: 5.3% (69/1311) were shared across all cell types; 56.3% (738/1311) were shared between a grouping of cell types; and 38.4% (504/1311) were cell-type-specific, with ductal cells having the most unique genes. For genes associated with glucose and time–glucose, the vast majority of associated genes were cell-type-specific, particularly in beta cells (94.8% [327/345] for glucose; 69.9% [292/418] for time–glucose).

We identified enriched gene sets for genes differentially expressed in the continuous models (ESM Fig. 16). We observed similar enriched processes as in the discrete models, including transcriptional regulation, stress response and protein synthesis pathways. In beta cells, similar gene sets were enriched across all three continuous models and largely featured terms related to metabolism, ATP synthesis, RNA processing and protein folding.

### Nomination of candidate effector genes for type 2 diabetes and type 2 diabetes-related traits

We sought to prioritise candidate effector genes for type 2 diabetes and type 2 diabetes-related traits by incorporating genetic association summary statistics. Using the PoPS method [29], we modelled genetic association summary statistics for type 2 diabetes, HbA_1c_, random blood glucose and fasting blood glucose using genomic features derived across all cell types in this study (e.g. cell-type-specific expression patterns, differential expression test statistics).

**Fig. 4:**
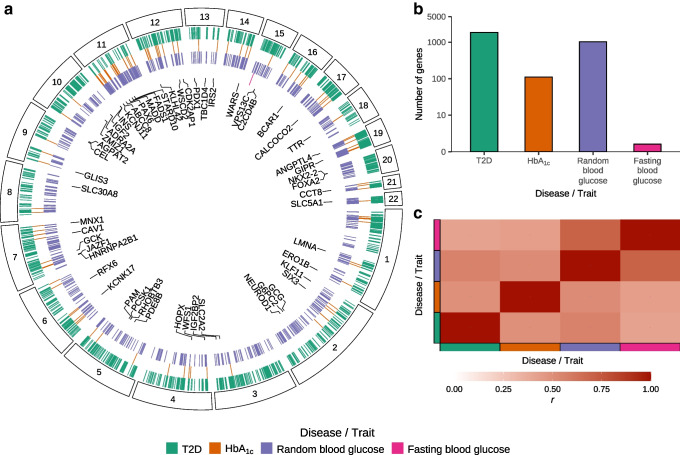
Candidate effector gene prediction. (**a**) Genomic locations for candidate effector genes (tick marks indicate FDR<5%) from genetic association summary statistics for type 2 diabetes (T2D), HbA_1c_, random blood glucose and fasting blood glucose (see key for colour code). Segments correspond to the chromosome location of genes (numerical labels). (**b**) Number of candidate effector genes (FDR<5%) across phenotypes. (**c**) Heatmap of *p* value correlations (red gradient) across phenotypes (see key for colour code)

We identified 2449 unique candidate effector genes across all four phenotypes (FDR<5%): 1855 for type 2 diabetes; 111 for HbA_1c_; 1023 for random blood glucose; and 1 for fasting blood glucose (Fig. 4). We compared the −log_10_(*p* value) for each gene across phenotypes and found them to be moderately correlated (minimum *r*=0.5883; Fig. 4c). Among the candidate effector genes, 1949 (79.6%) were associated with only one phenotype, 459 (18.7%) were associated with two phenotypes, 41 (1.7%) were associated with three phenotypes and none were associated with all four phenotypes. Of the 132 effector genes from the Type 2 Diabetes Knowledge Portal (https://t2d.hugeamp.org), we identified 48 (36.4%) as candidate effector genes for at least one phenotype, including *INS*, *PAX6*, *ABCC8*, *KCNJ11*, *NKX2–2*, *G6PC2*, *PAM*, *FOXA2*, *SLC30A8*, *RFX6*, *PCSK1* and *GLIS3*.

### Functional characterisation of candidate effector genes in a beta cell line

**Fig. 5:**
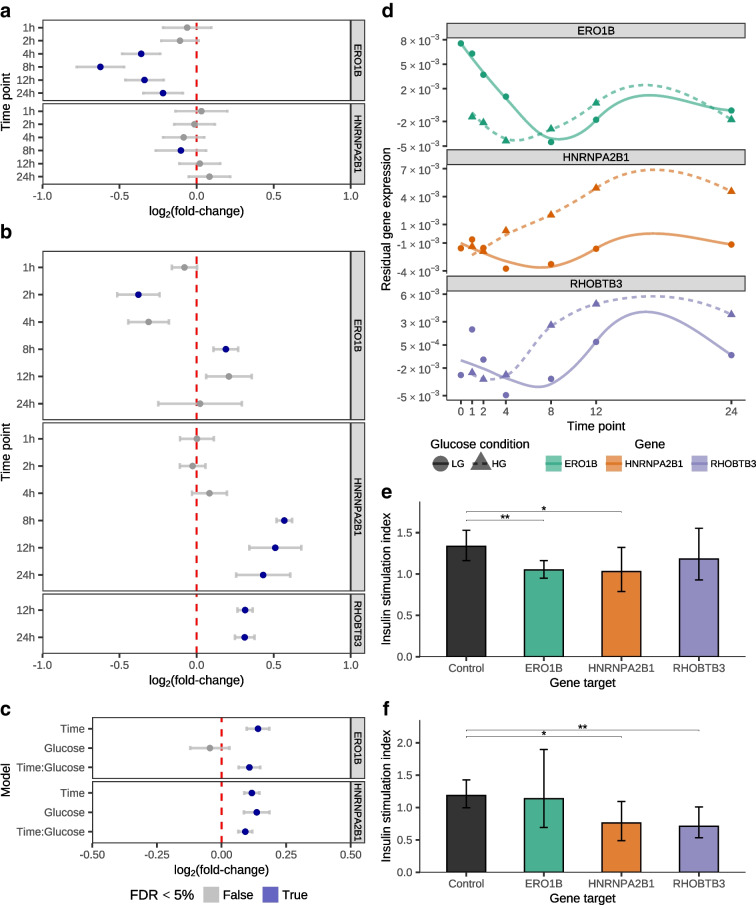
Functional validation of candidate effector genes. (**a**–**c**) Log_2_(fold-change) of candidate effector genes (facets) in the BvL (**a**) and LvH (**b**) and continuous time differential expression analyses (**c**) in beta cells. Error bars represent 95% CIs. Colour denotes FDR<5%. (**d**) Residual expression of candidate effector genes in beta cells at sampled time points under low and high glucose exposure (lines and points). (**e**, **f**) Insulin stimulation index from extracellular insulin after 1 h glucose exposure (**e**) and intracellular insulin after 24 h glucose exposure (**f**) across control and CRISPRi EndoC-βH1 experiments *p<0.05, **p<0.01 (Welch’s *t* test). Error bars represent 95% CIs

Using the EndoC-βH1 human beta cell line [31], we selected four candidate effector genes (*ERO1B*, *HNRNPA2B1*, *RHOBTB3* and *HOPX*; Fig. 5a–d), decreased the expression of these genes using CRISPRi, and characterised the effect on insulin production and secretion after glucose stimulation. We used qRT-PCR to compare expression of the targeted genes in the lines transfected with the CRISPRi constructs to control lines transfected with non-targeting guide RNAs. We confirmed decreased expression (*p*<0.05, Welch’s *t* test) for all genes apart from *HOPX* (ESM Fig. 17). Focusing on the genes that showed successful inhibition (i.e. *ERO1B*, *HNRNPA2B1*, *RHOBTB3*), we repeated the CRISPRi experiment, stimulated cells in high (15 mmol/l) and low (2.8 mmol/l) glucose concentrations for 24 h, and measured extracellular insulin content (i.e. insulin secretion) at 1 h and intracellular insulin content (i.e. insulin production) at 24 h, using non-targeting gRNAs as controls (ESM Fig. 18). For both measurements, we calculated the insulin stimulation index as the ratio of mean insulin content in high glucose conditions to the mean insulin content in low glucose conditions for downstream comparisons (Fig. 5e,f). Compared with controls, we found that knockdown of *ERO1B* and *HNRNPA2B1* decreased insulin secretion (i.e. lower extracellular insulin stimulation index at 1 h; *p*<0.05, Welch’s *t* test; Fig. 5e) and that knockdown of *HNRNPA2B1* and *RHOBTB3* decreased insulin production (i.e. lower intracellular insulin stimulation index at 24 h; *p*<0.05, Welch’s *t* test; Fig. 5f). Taken together with the functional assay results, our data implicates these genes as important regulators of insulin secretion and production upon glucose exposure in beta cells.

## Discussion

In this study, we present an in-depth characterisation of the 24 h transcriptomic response of human pancreatic islets to glucose exposure across cell types and time.

To characterise the effects of glucose exposure on islet cell types through time, we fit a variety of models. Across all cell types, we show that time in culture has a substantial impact on gene expression and, if not experimentally controlled for, will confound differential expression results (e.g. in the case of the BvH model). These findings are an important reminder that islet cell types, especially beta cells, are sensitive to intentional and unintentional experimental perturbations, as we have shown previously [46]. An important caveat is that the in vitro experimental conditions are not exact representations of in vivo conditions; factors such as time in culture can induce additional expression changes that must be controlled for. As an example, we found that many of the genes induced by glucose in beta cells also show time in culture effects (Fig. 3), suggesting that primary islets should be analysed as quickly as possible upon harvesting. In addition, previous studies have also identified single-cell dissociation protocols as another source of variation that can lead to increased expression of immediate early genes (e.g. *FOS*, *JUNB*) [47, 48]. Although we find several of these genes to be differentially expressed in multiple cell types across all models (ESM Table 4), it is difficult to disentangle dissociation-induced effects from time in culture or glucose-induced effects, as these genes play an important role in the islet response to glucose [49, 50]. Nonetheless, dissociation-induced stress is a potential confounder to be aware of in single-cell datasets such as the one presented in this study.

When we focus only on glucose-related effects after controlling for time, we find that the transcriptomic response in islets is primarily driven by beta cells. Within beta cells, we observe immediate transcriptional activity upon exposure to glucose. Transcriptional activity peaks, however, at 8 h and is largely maintained through to 24 h (e.g. in the LvH model, >65% of differentially expressed genes are from time points ≥8 h). The observed timing of the transcriptional response of beta cells to glucose stimulation underscores the importance of prolonged glucose exposure assays.

Our work builds on previous glucose stimulation transcriptomic studies in bulk islets [9, 10] by characterising the effects of glucose stimulation on individual islet cell types at multiple time points (ESM Fig. 19–22, ESM Table 5). From our high-resolution data, we identify specific cell types likely responsible for 305 (6.5%) of the genes previously identified in bulk studies, 260 of which are differentially expressed in beta cells only. For the remaining 45 genes, nine are cell type-specific and 36 are identified in multiple cell types. Notably, all of the genes identified in multiple cell types were found in beta cells too. However, many genes identified as differentially expressed were not shared between our study and previous ones. These differences could be due to a variety of reasons, including differences in islet preparation, differences in glucose stimulation concentrations, differences in the duration of glucose stimulation and confounding effects of cell type heterogeneity in bulk tissue studies [51].

By modelling genetic association summary statistics using genomic features derived from the single-cell data of this study, we nominate additional candidate effector genes for type 2 diabetes, HbA_1c_, random blood glucose and fasting blood glucose. We identify many genes with a well-defined role in relation to type 2 diabetes (e.g. *ABCC8*, *SLC30A8*), including 48 of the 132 type 2 diabetes effector genes from the Type 2 Diabetes Knowledge Portal. We also demonstrate the role of three candidate effector genes, *ERO1B*, *HNRNPA2B1* and *RHOBTB3*, in insulin production and secretion in a human beta cell line, building on previous knowledge about these genes. *ERO1B* (also known as *ERO1LB*), an endoplasmic reticulum stress gene responsible for protein folding in the secretory pathway [52], is highly expressed in the pancreatic islet [53] and has been shown to regulate intracellular and secreted insulin in rodent models [54, 55]. Our data from human beta cells replicates the secreted insulin effect but did not identify an intracellular insulin effect. *HNRNPA2B1* is an RNA-binding protein involved in many post-transcriptional RNA regulation processes [56, 57] that has been shown to be differentially expressed upon glucose exposure in human EndoC-βH1 cells [58] and to regulate intracellular and secreted insulin content in mouse insulinoma MIN6 cells [59]. Our results in EndoC-βH1 cells demonstrate that the *Hnrnpa2b1* loss of function effects on intracellular and secreted insulin content hold true in human cells. Last, *RHOBTB3* plays a central role in transporting secretory proteins from endosomes to the Golgi apparatus [60] and has been associated with type 2 diabetes in primary human islets [61] as well as palmitic acid exposure in EndoC-βH1 cells [61]. Although a previous study found no differences in *RHOBTB3* expression after up to 48 h glucose stimulation in EndoC-βH1 cells [61], our results demonstrate a dynamic expression pattern of *RHOBTB3* across 24 h under low and high glucose exposure (Fig. 5d) and establish a strong effect of *RHOBTB3* inhibition on intracellular insulin content in EndoC-βH1 cells after glucose exposure for 24 h.

In conclusion, an important approach to help determine the molecular drivers of type 2 diabetes pathogenesis and progression is by understanding the effects of diverse, disease-relevant, environmental exposures on gene expression in type 2 diabetes-relevant tissues such as pancreatic islets. In this study, we report the effects of sustained glucose exposure on gene expression in islet cell types. Though restricted to just a 24 h exposure and limited in sample size due to the intensive sampling across time and glucose exposures, these data may provide a relevant window into the consequences of the hyperglycaemic conditions that occur as one transitions from impaired glucose tolerance to type 2 diabetes.

## Supplementary Information

Below is the link to the electronic supplementary material.

Supplementary file1 (PDF 61477 KB)

Supplementary file2 (XLSX 25 KB)

## Data Availability

The scRNA-seq data from the 24 h glucose exposure experiment performed in this study are available in the database of Genotypes and Phenotypes (dbGap; https://www.ncbi.nlm.nih.gov/gap/) with accession no. phs001188.v3.p1. Study metadata and summary statistics for the differential expression, gene set enrichment and candidate effector gene prediction analyses are available in the Zenodo data repository (https://zenodo.org/) under accession number 11123248. The code used in this study is publicly available at https://github.com/CollinsLabBioComp/publication-islet_glucose_timecourse.

## Abbreviations

BH: Benjamini–Hochberg
BvH: Basal vs high
BvL: Basal vs low
CRISPRi: CRISPR interference
DGE: Differential gene expression
FDR: False discovery rate
GO: Gene ontology
gRNA: Guide RNA
LvH: Low vs high
PC: Principal component
PoPS: Polygenic priority score
qRT-PCR: Quantitative RT-PCR
scRNA-seq: Single-cell RNA-seq
UMI: Unique molecular identifier
