# Supplementary material for: Single-cell transcriptomic profiling of human pancreatic islets reveals genes responsive to glucose exposure over 24 h
Source: Diabetologia. 2024 Jul 5;67(10):2246–59. doi: 10.1007/s00125-024-06214-4 (PMC11447040; doi:10.1007/s00125-024-06214-4)
Supplement: Supplementary file 1 — Supplementary file1 (PDF 61477 KB) [file 125_2024_6214_MOESM1_ESM.pdf]

Supplemental Materials and Methods

Islet procurement and processing

We obtained purified human pancreatic islets from two individuals through Prodo Laboratories (Aliso Viejo, CA; ESM Table 1). The purified islets were cultured in PIM(S) complete media (Prodo Laboratories, Aliso Viejo, CA) at a density of 10,000 islet equivalents (IEQs) per $150mm^2$ for 72 hours at 37ºC. Islets were packaged and transported to our laboratory at 4ºC over a period of 24 hours. Upon receipt, we equilibrated the islets to 37ºC for 1 hour in 2.8 mmol/l glucose media prior to downstream processing.

Prior to shipment, islets were characterised by Prodo Laboratories using a glucose-stimulated insulin release assay (ESM Fig. 1). Briefly, islets were washed twice in G55 3 mmol/l media (50/50 Hams F10/DMEM without glucose, 7.14 mmol/l $NaHCO_3$, 0.75 mmol/l $CaCl_2$, 7.4 pH) and resuspended in 3 mmol/l media with 2.5% BSA. Seventy-five IEQs were added to each of three wells (of 24-well cell culture plate) containing Millicell cell culture inserts (12 μm pore, Millipore-Sigma Cat. #PIXP01250) and incubated overnight at 37ºC with 5% CO_2. Wells were washed twice with 3 mmol/l media, resuspended in 1 ml of fresh 3 mmol/l media, and incubated for one hour at 37ºC. Media was replaced with 1 ml fresh 3 mmol/l and incubated for a second hour. The assay began with a fresh media exchange of 1 ml of 3 mmol/l media and an incubation for one hour at 37ºC. Next, media was collected and exchanged with 3 mmol/l media supplemented with D-glucose to 15 mmol/l; cells were incubated at 37ºC for one hour. Media was collected and exchanged with 3 mmol/l media supplemented with D-glucose to 28 mmol/l; cells were incubated at 37ºC for one hour. Media was collected and exchanged with 3 mmol/l media supplemented with D-glucose to 28 mmol/l plus 0.1 mmol/l IBMX; cells were incubated at 37ºC for one hour. Media was collected and cells were washed twice with 3 mmol/l media, resuspended in fresh 3 mmol/l media, and incubated at 37ºC for one hour. Media was collected; cells were washed with 1 ml TE and resuspended in 1 ml TE buffer. Subsequently, islet cells were centrifuged at 500 g for 2 minutes and resuspended in 1 ml TE with 1% Triton X100 plus protease Inhibitor cocktail (Sigma-Aldrich Cat. #P2714). Each collected media and islet lysates were frozen at -20ºC. Insulin was measured in the collected

supernatants using an insulin enzyme-linked immunosorbent assay (ELISA; Mercodia Cat. #10-1113-01) according to the manufacturer instructions. Finally, islet lysates were used for insulin content measurement by ELISA and DNA quantification for normalisation.

Genotyping

We transferred 500-1,000 IEQs to tissue culture-treated flasks, cultured them as in Gershengorn et al. [1], and isolated genomic DNA for genotyping. We genotyped isolated DNA at the National Human Genome Research Institute (NHGRI) genomics core facility using the InfiniumOmni2-5Exome-8 BeadChip array, v1.3 (HP18227) or v1.5 (HP19208; Illumina, San Diego, CA, USA). We mapped Illumina array probe sequences to the GRCh37 (hg19) genome assembly using novoalign v2.07.11 (http://www.novocraft.com/products/novoalign). Subsequently, we excluded single nucleotide polymorphisms (SNPs) with (i) ambiguous probe alignments, (ii) a 1000 Genomes (1000G) phase 3 variant with a minor allele frequency (MAF) ≥1% within 7 base pairs (bp) of the 3' end of the probe, or (iii) call rates <95%. After these filters, we performed imputation using Minimac4 v1.7.3 [2] on the TOPMed Imputation Server [3] with the TOPMed-r2 reference panel [4] (we note that this step included GRCh38 liftover). For subsequent analysis, we removed SNPs with an imputation $r^2 \leq 0.3$.

Single-cell RNA sequencing of glucose-stimulated pancreatic islets

We exposed pancreatic islets to either low (2.8 mmol/l) or high (15 mmol/l) glucose for 24 hours and performed single-cell RNA sequencing (scRNA-seq) at 1, 2, 4, 8, 12, and 24 hour time points. In addition, we performed scRNA-seq on islets at baseline 2.8 mmol/l glucose prior to starting the stimulation experiment (Islet procurement and processing section), which we label as time point 0. To perform the glucose stimulation assay, we plated aliquots of 2,000 IEQs from each donor into four wells of a 12-well plate for each time point (6 plates per donor; two wells for 0 hours since no high glucose exposure) with either low or high glucose in KREBS solution. We incubated the islets at 37°C for the duration of the experiment and sampled wells at their respective time points for scRNA-seq (0, 1, 2, 4, 12, and 24 hours for donor HP18227; 0, 2, 4, 8, 12, and 24 hours for

donor HP19208). Each well corresponded to a separate scRNA-seq experiment, resulting in two replicates for each donor, time point, and glucose condition.

For each sampled time point, we dissociated the islet aliquot and performed scRNA-seq. To dissociate the islets, we incubated the 2,000 IEQ aliquot in 1 ml Accutase solution (Innovation Cell Technologies, Inc) at 37ºC for 10 minutes, washed them with 2 ml PIM(S)TM (Prodo Islet Media, Prodo Laboratory Inc, Irvine CA), incubated the islets for 10 minutes at 37ºC in 2 ml PBS with 4U Dispase I (Roche Diagnostics) / 2U DNase I (ThermoFisher Scientific), washed them once, and resuspended them in PIM(S). We then passed the final cell suspension through a BD 40 mm cell strainer to remove aggregates and assessed the cells for viability and abundance via staining with acridine orange and DAPI (Chemometec Nucleocounter NC-3000). With the filtered suspension, we generated a single-cell mRNA library using either a 10X Genomics SC3'v2 or SC3'v3 chemistry kit according to the manufacturer's instructions (ESM Table 1). We quantified the barcoded sequencing library with the Quant-IT PicoGreen dsDNA kit (P11496, Invitrogen), diluted cells at 3 nM, and sequenced them on an Illumina HiSeq3000 machine. Across all libraries, we sequenced at an average of 296 million reads per sample.

Single-cell RNA-seq processing and quality control

We used CellRanger v3.1.0 with default parameters to process and align reads to GRCh38.p13 with Ensembl version 98 transcript definitions (reference file distributed by 10X Genomics), identify cell-containing droplets, and generate cell $\times$ gene count matrices.

Next, we used DecontX [5] (implemented in celda v1.14.0 [6]) to (i) remove droplets with a high ambient transcript contamination from the single-cell sequencing experiment and (ii) adjust the raw counts matrix for the ambient expression signature. DecontX requires a gene count matrix of droplets that likely do not contain cells (i.e., "empty droplets"), a count matrix of droplets that likely contain cells, and cell cluster labels. We used the empty droplet count matrix generated by CellRanger, the cell count matrix generated by CellRanger, and cell

type labels derived from clustering the cell count matrix with the Seurat v4.3.0 [7] single-cell analysis workflow with default parameters, except for using 20 principal components for nearest neighbour calculations. We applied DecontX with default parameters apart from setting the delta parameters to 10 and 30, which represent the prior expectations for the proportion of native and contamination counts, respectively. We removed cell-containing droplets (defined by CellRanger) with >10% ambient contamination as estimated by DecontX from the original CellRanger gene count matrix. Next, we re-ran the DecontX workflow (including Seurat-derived cell type labelling) using the same data and settings apart from removing cells with >10% ambient contamination from the CellRanger cell count matrix. To generate the final cell $\times$ gene count matrices, we applied the same ambient contamination filter (removing droplets with >10% ambient contamination) and used the DecontX-adjusted count matrix (ESM Fig. 2A).

We identified and removed multiplets using scrublet v0.2.1 [8], simulating 100,000 multiplets and calculating the multiplet threshold using the threshold_li function from the scikit-image package v0.18.1 [9], initialised using the threshold_otsu function. We removed low-quality cells, defined as cells in which the percentage of counts originating from the mitochondrial genome was >50%. Next, we used an isolation forest (scikit-learn v0.23.2) to remove outlier cells based on (i) the total number of unique molecular identifier (UMI) counts per cell and (ii) the number of genes expressed (≥1 count) per cell. Finally, we verified the identity of each sample and checked for DNA contamination by comparing the sequence reads to genotypes from both donors using VerifyBamID v1.1.3 [10].

For subsequent analyses, we further processed the data using scanpy v1.6.0 [11]. We removed genes that were expressed (≥1 count) in ≤5 cells across the whole dataset (sc.pp.filter_genes with min_cells=5) as well as mitochondrial and ribosomal genes. To account for variable sequencing depth across cells, we normalised UMI counts by the total number of counts per cell, scaled to counts per 10,000 (CP10K; sc.pp.normalise_per_cell), and log-transformed the CP10K expression matrix (ln[CP10K+1]; sc.pp.log1p).

As a final quality control step, we calculated the gene expression correlation between replicates for each donor. Using the cell type annotations (Cell type annotation section), we generated pseudobulk data for each donor replicate, cell type, time point, and glucose condition. We normalised the gene counts to total counts and calculated the Spearman's correlation coefficient for each donor replicate, cell type, time point, and glucose condition (ESM Fig. 3).

Cell type annotation

Prior to calculating cell type clusters, we reduced the cell ✕ gene expression matrix to a core set of independent variables using principal component analysis (PCA). First, we selected the 2,000 most variable genes across samples by (i) calculating the most variable genes per sample and (ii) selecting the 2,000 genes that occurred most often across samples (sc.pp.highly_variable_genes with flavor='seurat' and batch_key=sample). After mean centering and scaling the ln(CP10K+1) expression matrix to unit variance, we performed principal component analysis (PCA; sc.tl.pca) using the 2,000 most variable genes. To select the number of PCs for subsequent analyses, we used a scree plot [12] and calculated the "knee/elbow" derived from the variance explained by each PC using the kneedle estimator v0.7.0 [13], selecting 9 PCs. Finally, we used harmony v0.0.5 [14] with default parameters to integrate samples and control for sample-specific batch effects prior to clustering.

Using the harmony-adjusted PCs, we calculated clusters using the Leiden graph-based clustering algorithm v0.8.3 [15] (sc.tl.leiden) with default parameters. We generated clusters across a wide range of resolutions to empirically determine the optimal clustering resolution. For each resolution considered, we divided the data into training (2/3 of cells) and test (1/3 of cells) sets. Using the training data, we fit a single layer dense neural network, implemented in keras v2.4.3, to predict cluster identity from the expression of all genes (ln[CP10K+1]). Within the test set, we predicted the cluster label of each cell and calculated the Matthew's correlation coefficient (MCC) for each cluster, a metric that robustly summarises all four confusion matrix categories: true positives, false negatives, true negatives, and false positives [16]. For the final cluster

classifications, we chose the largest resolution where the minimum MCC across all clusters was >0.75, selecting a resolution of 0.25 (8 clusters, ESM Fig. 2B-C).

To determine the cell type identity of clusters, we used well-established marker genes for the cell types common to islets (ESM Fig. 2D): *GCG* (alpha cells), *INS* (beta cells), *PPY* (gamma cells), *SST* (delta cells), *PRSS1* (acinar cells), *KRT19* (ductal cells), *COL1A1* (stellate cells), and *CD68* (macrophages). To visualise the clusters, we performed dimensionality reduction using the uniform manifold approximation and projection (UMAP) algorithm, implemented within scanpy (sc.tl.umap) with default parameters, except for changing the minimum distance and spread parameters from 0.5 to 1.0.

Finally, in order to more completely capture the heterogeneity in expression profiles of each cell type, we re-introduced cells removed during the quality control procedures (e.g., cells filtered due to a large percentage of mitochondrial reads) for subsequent analysis when we could predict the cell type confidently. Specifically, using the cell type classifier trained during cluster resolution optimization, we predicted the cell type identity of filtered cells and added cells back into the dataset when the maximum probability across all cell type predictions for a given cell was >0.5 (meaning the probability of a cell mapping to a specific cell type was greater than the sum of all other cell type probabilities). In total, this procedure added back 18,112 additional cells.

Time interpolation

We derived interpolated time from sampled time points (t=1h, 2h, 4h, …, 24h) to better model the cellular state of each cell at each time point, assuming that cells captured at each time point are not at a uniform cellular state in their response to time in culture and/or glucose exposure but rather are distributed across cellular response phases. Prior to calculating interpolated time, we removed the 0 hour time point as there was no high glucose treatment at this time point. For each cell type, donor, and glucose condition, we calculated the *n* nearest

neighbours based on gene expression values (ln[CP10K+1]) using scvelo v0.2.4 [17] (scvelo.pp.neighbors) and derived interpolated time, $\hat{t}$, for each cell using the following formula:

$$\hat{t} = t * w + \frac{\sum_{i}^{n} d_i^{-1} t_i}{\sum_{i}^{n} d_i^{-1}} * (1 - w)$$

where t is the target cell's sampled time point, w is a weight coefficient for t, d_i is the distance of the target cell to neighbouring cell i, and t_i is the sampled time point of neighbouring cell i. To select values for n and w, we performed an exhaustive grid search to evaluate the effect of all combinations of n and w on the stability of $\hat{t}$, where n ranged from 0 to 150 in intervals of 5 and w ranged from 0 to 1 in intervals of 0.25. For each value of w, we considered all values of n and compared $\hat{t}$ to $\hat{t}$ derived from the previous n value using mean squared error (MSE) as a stability metric (ESM Fig. 12A). Across all values of w, we found $\hat{t}$ was stable at $n \geq 75$ and selected a value of $n=75$ for further analyses. To determine the value of w, we set $n=75$, calculated $\hat{t}$ for w values running from 0 to 1 in intervals of 0.25, standardised $\hat{t}$, and evaluated the effect of different $\hat{t}$ derivations on the differential gene expression results for the time-glucose interaction model (Differential gene expression analysis section), comparing the signed -$\log_{10}$(P-values) for each $\hat{t}$ derivation to the results of using t (the sampled time point; ESM Fig. 12B). We found the results were highly correlated across all values of w. For subsequent analysis, we selected a value of 0.25 for w since the results were extremely concordant with t, yet showed a slight increase in power. We calculated the final interpolated time values using $n=75$ and $w=0.25$ and standardised interpolated time values within each glucose condition, donor, and cell type to a 0-1 scale.

Differential gene expression analysis

For each cell type, we performed differential gene expression (DGE) analysis (both discrete time point DGE models and continuous time DGE models) using MAST v1.20.0, a two-part, generalised linear model with a logistic regression component for the discrete process (i.e., a gene is expressed or not) and linear regression component for the continuous process (i.e., the expression level) [18]. Briefly, for gene i, and cell k, let Z_{ki}

indicate whether gene i is expressed in cell k and Y_{ki} denote the ln(CP10K+1) normalised gene expression. We tested for association using the two-part regression model:

$$logit(Pr(Z_{ki} = 1 \mid X_k)) = X_k \beta_i \quad (1)$$

$$Pr(Y_{ki} = y \mid Z_{ki} = 1) = N(X_k \beta_i, \sigma_i^2) \quad (2)$$

where X_k are the predictor variables for cell k and β_i is the vector of fixed effect regression coefficients.

Across all DGE models, we included cell complexity (i.e., the number of genes detected per cell) as a fixed effect covariate to control for unobserved nuisance variation (e.g., cell size) [18]. We included participant and experiment identifiers as random effects to control for pseudoreplication bias [19]. We also included additional fixed effect variables depending on the specific model (see subsequent paragraphs).

For the discrete time DGE models, we fit separate models for each cell type and time point. We considered three models. First, in the "basal-versus-high" (BvH) model, we compared basal cells (0 hours, 2.8 mmol/l glucose) to cells exposed to high glucose (15 mmol/l) across time (e.g., basal versus 1 hour of high glucose exposure, basal versus 2 hours of high glucose exposure) by including a fixed effect "glucose status" variable (0 = basal/low, 1 = high). Second, in the "basal-versus-low" (BvL) model, we compared basal cells (0 hours, 2.8 mmol/l glucose) to cells maintained in low glucose (2.8 mmol/l) across time (e.g., basal versus 1 hour of low glucose exposure, basal versus 2 hours of low glucose exposure) by including a fixed effect "time point" variable (0 = basal at 0 hours, 1 = other time points). Third, in the "low-versus-high glucose" (LvH) model, we compared cells exposed to low glucose (2.8 mmol/l) to cells exposed to high glucose (15 mmol/l) at each time point (e.g., 1 hour of low glucose exposure versus 1 hour of high glucose exposure, 2 hours of low glucose exposure versus 2 hours of high glucose exposure) by including a fixed effect "glucose status" variable (0 = low, 1 = high).

For the continuous time DGE models, we fit separate models for each cell type, jointly analysing cells across all glucose exposures and time points, excluding the 0 hour time point. We considered three different models: (i) a “continuous time” model to test for time effects, (ii) a “glucose” model to test for glucose effects, and (iii) a “time-glucose interaction” model to test for an interaction effect between time and glucose concentration. For all three models, we included glucose concentration (0 = low, 1 = high) and continuous time as fixed effect variables (in addition to cell complexity and participant identifiers). For the “time-glucose interaction” model, we used the same model but added an additional time-glucose concentration interaction term.

Finally, for each model, we controlled for the number of tests performed with each cell type using the Benjamini-Hochberg procedure [20] and *P*-values obtained from the hurdle model, derived from the summed χ^2 null distributions of the discrete (Z_i) and continuous (Y_i) components, as described in Finak et al. [18]. To increase the speed of each test, prior to fitting models for each cell type, we removed genes with a median CP10K<1. To visualise gene expression patterns across time points, we plotted the residual gene expression after adjusting the ln(CP10k+1) normalised gene expression for cell complexity, participant, and experiment using lme4 v1.1-35.1 [21], modelling cell complexity as a fixed effect and participant and experiment identifiers as random effects.

Gene ontology enrichment and clustering analysis

For each differential gene expression model (e.g., BvL at 1 hour, BvL at 2 hour, continuous time, glucose), we calculated the enrichment of differentially expressed genes (FDR<5%) in gene ontology (GO) terms from the “biological process” ontology using the compareCluster function from clusterProfiler v4.8.3 [22] with “enrichGO” as the enrichment function (i.e., the “fun” parameter in compareCluster). We controlled for the number of tests performed using the Benjamini-Hochberg procedure [20].

To visualise enrichment results (FDR<5%), we used the emapplot function from enrichplot v1.20.3 [23] to create networks where vertices depict GO terms and edges connect similar terms. To model the semantic similarity between GO terms, we used the godata function from GOSemSim v2.26.1 [24] and calculated the similarity matrix with the pairwise_termsim function from enrichplot, setting the method parameter to "Rel". From the resulting similarity matrix, we generated the plots using the emapplot function and the following options: node_label="group", edge.params=list(min=0.75), pie.params=list(pie="Count"), and cluster.params=list(cluster=T). In the plots generated with these settings, edges connect GO terms (vertices) with a similarity ≥0.75, vertices are pie charts representing the relative proportion of the number of genes within a GO term, and terms are grouped into larger clusters based on their shared, semantic similarity (clusters calculated with kmeans function from stats v4.3.2 where k=sqrt[number_vertices] as implemented in emapplot). For the final plots, we labelled each group of terms based on the GO terms within each group manually.

Nomination of candidate effector genes for type 2 diabetes and type 2 diabetes-related traits

We nominated candidate effector genes using the Polygenic Priority Score (PoPS) method v0.2 [25]. Briefly, the PoPS method requires gene-level association statistics, calculated from a genetic association study, and a genomic feature matrix across genes (gene ✕ feature matrix). Following the PoPs workflow, we generated gene-level statistics using MAGMA v1.10 [26], with the window option set to "10,10". As input to MAGMA, we used (i) gene locations released in the PoPS repository (accessed via https://github.com/FinucaneLab/pops), (ii) the 1000 Genomes Phase 3 European linkage disequilibrium reference panel (accessed via https://ctg.cncr.nl/software/magma), and (iii) publicly available summary statistics for type 2 diabetes [27], fasting blood glucose [28], random blood glucose [27], and glycated hemoglobin [27]. Next, we constructed a "control" and "test" genomic feature matrix. For the "control" matrix, we encoded housekeeping genes [29, 30] using a binary system (1=housekeeping gene, 0=all other genes). For the "test" matrix, we generated features from the single-cell data presented in this study as performed in Weeks et al. [25], including (i) principal component gene weights across all cells and within cells for each cell type, (ii) test statistics for cell type marker genes (Welch's *t*-test [31]; including the test statistic and a binary indicator for up- and down- regulated genes

with FDR<5%), and (iii) average expression within each cell type. In addition, we also included (i) cell type gene expression specificity values calculated using CELLEX v1.2.2 [32] and (ii) test statistics from all models but the BvH model (Differential gene expression analysis section, including the test statistic and a binary indicator for up- and down- regulated genes with FDR<5%). To calculate *P*-values, we permuted the "test" feature matrix gene identifiers 1,000 times and re-ran PoPS. For each gene, we calculated empirical *P*-values, defined as $(r+1) / (n+1)$, where r is the number of permuted PoP scores greater than the observed and n is the total number of permutations [33]. Finally, we controlled the false discovery rate across all genes considered using the Benjamini-Hochberg procedure [20].

CRISPR interference experiments

We obtained EndoC-βH1 cells [34] (RRID: CVCL_L909, female) from the Centre National de la Recherche Scientifique (CNRS) and cultured the cells in DMEM containing 5.6 mmol/l glucose, 2% BSA (Sigma-Aldrich), 50 µmol/l 2-mercaptoethanol (Thermo Fisher Scientific), 10 mmol/l nicotinamide (Sigma-Aldrich), 5.5 µg/ml transferrin (Sigma-Aldrich), 6.7 ng/ml selenite (Sigma-Aldrich), 100 U/ml penicillin and 100 µg/ml streptomycin. To perturb the transcriptional expression of candidate genes, we designed three different guide RNAs (gRNAs) targeting the regions near the transcription start site (TSS) of each candidate gene, using the web resources available at http://chopchop.cbu.uib.no (ESM Table 2). We synthesised gRNAs targeting candidate genes and two non-targeting gRNAs (that do not recognize any sequence in the human genome [35]), as top- and bottom-strand oligos (IDT) and cloned them into the BsmBI-digested CRISPR interference (CRISPRi) vector (Addgene, #139097) according to the previously described instructions [36]. To produce the lentivirus expressing each CRISPRi system (dCas9-KRAB + gRNA), we transfected HEK293T cells with lentivirus packaging plasmids pMD2.G and psPAX2 (Addgene, #12259 and #12260) along with either CRISPRi plasmid targeting each candidate gene or the non-targeting control plasmids. We concentrated the viral suspension (collected at 48 hours and 72 hours post-transfection) using the Lenti-X Concentrator (Takara Bio) and resuspended the viral particles with DMEM in 1/20 of the original volume (20X concentration). For transduction, we seeded around 1 million EndoC-βH1 cells in six-well

plates and infected them with 0.2 ml of viral suspension supplemented with polybrene at a final concentration of 8 μg/mL. After 72 hours post-transduction, we selected for infected EndoC-βH1 cells by exposing the cells to 2 μg/mL puromycin for one week, which we used for subsequent functional assays.

Quantitative reverse transcription polymerase chain reaction experiments

We isolated total RNA from EndoC-βH1 cells using the RNeasy Plus Mini Kit (QIAGEN), quantified RNA with a microplate spectrophotometer (Agilent), and synthesised cDNA with a high-capacity reverse transcription kit (Thermo Fisher Scientific). We performed real-time qPCR with a LightCycler 480 (Roche) instrument with LightCycler DNA master SYBR Green I reagents (Roche). We listed the sequences of qRT-PCR primers specific to each candidate gene and the reference gene (*GAPDH*) in ESM Table 3. We determined the Delta-delta-cycle threshold (DDCT) relative to the *GAPDH* and calculated the mean and standard deviation for each group using four independent biological replicates.

Glucose-stimulation experiment of CRISPRi EndoC-βH1 cells

We seeded 1 million CRISPRi cells and the control cells in 12-well plates and let them recover for 2 days before the glucose exposure experiments. We removed the medium, washed the cells with a fresh KRBH buffer, and fasted the cells in 1 ml low glucose (LG) KRBH (i.e., KRBH with 2.8 mmol/l glucose) at 37°C for 1 hour. We then aspirated the LG KRBH buffer and added 1.2 ml of either LG KRBH or high glucose (HG) KRBH (i.e., KRBH with 15 mmol/l glucose) in each well, generating at least three biological replicates per glucose condition. After incubating cells at 37°C for 1 hour, we spun the plate at 300g for 5 minutes, collected the top 200 μl supernatant from each well, and put the cells back in the 37°C incubator. After incubating cells in LG or HG KRBH for 24 hours, we removed the residual medium and dissociated cells from each well using the TrypLE Express reagents (Thermo Fisher Scientific). We quantified the number of cells in each well using an Automated Cell Counter (Thermo Fisher Scientific) and lysed them with 150 ul RIPA buffer (Sigma-Aldrich) supplemented with Protease and Phosphatase Inhibitor Cocktail (Thermo Fisher Scientific). We measured insulin content in the supernatant samples (collected at 1 hour after glucose exposure) and the cell lysis samples

(collected at 24 hours after glucose exposure) using the STELLUX Chemi Human Insulin ELISA Jumbo kit (Alpco) and calculated the insulin stimulation index as the ratio of the mean insulin content in the high glucose condition compared to the low glucose condition. In the 24-hour experiment, we normalised insulin content by the number of cells to account for potential cell death during the 24-hour glucose exposure process. We used Fieller's method [37, 38] to calculate the 95% confidence interval of the high:low means, which in some instances may be asymmetric [39], and compared the gene-targeting CRISPRi experiments to the control experiments using Welch's *t*-test [31].

Comparison with results of previous bulk islet transcriptome studies

Previous bulk islet transcriptomic studies report genes associated with glucose stimulation using a study design similar to the LvH model of this study [40–42]. We compared the results of all glucose-relevant models (discrete models: LvH all time points; continuous models: glucose, time-glucose interaction) to studies where complete lists of differentially expressed genes were available: Ottosson-Laakso et al. [41] (5.5 mmol/l vs. 18.9 mmol/l glucose for 24 hours in normoglycemic and hyperglycemic donors) and Hall et al. [42] (5.6 mmol/l vs. 19 mmol/l for 48 hours). The results from these comparisons are recorded in ESM Fig. 19-22 and ESM Table 5.

Supplemental References

1. Gershengorn MC, Hardikar AA, Wei C, et al (2004) Epithelial-to-mesenchymal transition generates proliferative human islet precursor cells. Science 306:2261–2264. https://doi.org/10.1126/science.1101968
2. Fuchsberger C, Abecasis GR, Hinds DA (2015) minimac2: faster genotype imputation. Bioinformatics 31:782–784. https://doi.org/10.1093/bioinformatics/btu704
3. Das S, Forer L, Schönherr S, et al (2016) Next-generation genotype imputation service and methods. Nat Genet 48:1284–1287. https://doi.org/10.1038/ng.3656
4. Taliun D, Harris DN, Kessler MD, et al (2021) Sequencing of 53,831 diverse genomes from the NHLBI TOPMed Program. Nature 590:290–299. https://doi.org/10.1038/s41586-021-03205-y
5. Yang S, Corbett SE, Koga Y, et al (2020) Decontamination of ambient RNA in single-cell RNA-seq with DecontX. Genome Biol 21:57. https://doi.org/10.1186/s13059-020-1950-6
6. Wang Z, Yang S, Koga Y, et al (2022) Celda: a Bayesian model to perform co-clustering of genes into modules and cells into subpopulations using single-cell RNA-seq data. NAR Genom Bioinform

4:lqac066. https://doi.org/10.1093/nargab/lqac066

7. Hao Y, Hao S, Andersen-Nissen E, et al (2021) Integrated analysis of multimodal single-cell data. Cell 184:3573-3587. https://doi.org/10.1016/j.cell.2021.04.048

8. Wolock SL, Lopez R, Klein AM (2019) Scrublet: Computational Identification of Cell Doublets in Single-Cell Transcriptomic Data. Cell Syst 8:281-291.e9. https://doi.org/10.1016/j.cels.2018.11.005

9. van der Walt S, Schönberger JL, Nunez-Iglesias J, et al (2014) scikit-image: image processing in Python. PeerJ 2:e453. https://doi.org/10.7717/peerj.453

10. Jun G, Flickinger M, Hetrick KN, et al (2012) Detecting and estimating contamination of human DNA samples in sequencing and array-based genotype data. Am J Hum Genet 91:839–848. https://doi.org/10.1016/j.ajhg.2012.09.004

11. Wolf FA, Angerer P, Theis FJ (2018) SCANPY: large-scale single-cell gene expression data analysis. Genome Biol 19:15. https://doi.org/10.1186/s13059-017-1382-0

12. Cattell RB (1966) The scree test for the number of factors. Multivariate Behav Res 1:245–276. https://doi.org/10.1207/s15327906mbr0102_10

13. Satopaa V, Albrecht J, Irwin D, Raghavan B (2011) Finding a “kneedle” in a haystack: detecting knee points in system behavior. In: 2011 31st International Conference on Distributed Computing Systems Workshops. IEEE, pp 166–171

14. Polański K, Young MD, Miao Z, et al (2020) BBKNN: fast batch alignment of single cell transcriptomes. Bioinformatics 36:964–965. https://doi.org/10.1093/bioinformatics/btz625

15. Traag VA, Waltman L, van Eck NJ (2019) From Louvain to Leiden: guaranteeing well-connected communities. Sci Rep 9:5233. https://doi.org/10.1038/s41598-019-41695-z

16. Chicco D, Jurman G (2020) The advantages of the Matthews correlation coefficient (MCC) over F1 score and accuracy in binary classification evaluation. BMC Genomics 21:6. https://doi.org/10.1186/s12864-019-6413-7

17. Bergen V, Lange M, Peidli S, et al (2020) Generalizing RNA velocity to transient cell states through dynamical modeling. Nat Biotechnol 38:1408–1414. https://doi.org/10.1038/s41587-020-0591-3

18. Finak G, McDavid A, Yajima M, et al (2015) MAST: a flexible statistical framework for assessing transcriptional changes and characterizing heterogeneity in single-cell RNA sequencing data. Genome Biol 16:278. https://doi.org/10.1186/s13059-015-0844-5

19. Zimmerman KD, Espeland MA, Langefeld CD (2021) A practical solution to pseudoreplication bias in single-cell studies. Nat Commun 12:738. https://doi.org/10.1038/s41467-021-21038-1

20. Benjamini Y, Hochberg Y (1995) Controlling the false discovery rate: A practical and powerful approach to multiple testing. Journal of the Royal Statistical Society Series B (Methodological) 57:289–300

21. Bates D, Mächler M, Bolker B, Walker S (2015) Fitting linear mixed-effects models using lme4. J Stat Softw 67:1–48. https://doi.org/10.18637/jss.v067.i01

22. Wu T, Hu E, Xu S, et al (2021) clusterProfiler 4.0: A universal enrichment tool for interpreting omics data. Innovation (Camb) 2:100141. https://doi.org/10.1016/j.xinn.2021.100141

23. Yu G (2018) enrichplot: Visualization of Functional Enrichment Result. Bioconductor.

https://doi.org/10.18129/b9.bioc.enrichplot

24. Yu G, Li F, Qin Y, et al (2010) GOSemSim: an R package for measuring semantic similarity among GO terms and gene products. Bioinformatics 26:976–978. https://doi.org/10.1093/bioinformatics/btq064

25. Weeks EM, Ulirsch JC, Cheng NY, et al (2023) Leveraging polygenic enrichments of gene features to predict genes underlying complex traits and diseases. Nat Genet 55:1267–1276. https://doi.org/10.1038/s41588-023-01443-6

26. de Leeuw CA, Mooij JM, Heskes T, Posthuma D (2015) MAGMA: generalized gene-set analysis of GWAS data. PLoS Comput Biol 11:e1004219. https://doi.org/10.1371/journal.pcbi.1004219

27. Zhang MJ, Hou K, Dey KK, et al (2022) Polygenic enrichment distinguishes disease associations of individual cells in single-cell RNA-seq data. Nat Genet 54:1572–1580. https://doi.org/10.1038/s41588-022-01167-z

28. Manning AK, Hivert M-F, Scott RA, et al (2012) A genome-wide approach accounting for body mass index identifies genetic variants influencing fasting glycemic traits and insulin resistance. Nat Genet 44:659–669. https://doi.org/10.1038/ng.2274

29. Hounkpe BW, Chenou F, de Lima F, De Paula EV (2021) HRT Atlas v1.0 database: redefining human and mouse housekeeping genes and candidate reference transcripts by mining massive RNA-seq datasets. Nucleic Acids Res 49:D947–D955. https://doi.org/10.1093/nar/gkaa609

30. Gu J, Dai J, Lu H, Zhao H (2023) Comprehensive Analysis of Ubiquitously Expressed Genes in Humans from A Data-driven Perspective. Genomics Proteomics Bioinformatics 21:164–176. https://doi.org/10.1016/j.gpb.2021.08.017

31. Welch BL (1947) The generalization of 'student's' problem when several different population variances are involved. Biometrika 34:28–35. https://doi.org/10.1093/biomet/34.1-2.28

32. Timshel PN, Thompson JJ, Pers TH (2020) Genetic mapping of etiologic brain cell types for obesity. eLife 9:. https://doi.org/10.7554/eLife.55851

33. North BV, Curtis D, Sham PC (2002) A note on the calculation of empirical P values from Monte Carlo procedures. Am J Hum Genet 71:439–441. https://doi.org/10.1086/341527

34. Ravassard P, Hazhouz Y, Pechberty S, et al (2011) A genetically engineered human pancreatic β cell line exhibiting glucose-inducible insulin secretion. J Clin Invest 121:3589–3597. https://doi.org/10.1172/JCI58447

35. Doench JG, Fusi N, Sullender M, et al (2016) Optimized sgRNA design to maximize activity and minimize off-target effects of CRISPR-Cas9. Nat Biotechnol 34:184–191. https://doi.org/10.1038/nbt.3437

36. Legut M, Daniloski Z, Xue X, et al (2020) High-Throughput Screens of PAM-Flexible Cas9 Variants for Gene Knockout and Transcriptional Modulation. Cell Rep 30:2859-2868.e5. https://doi.org/10.1016/j.celrep.2020.02.010

37. Fieller EC (1940) The biological standardization of insulin. Supplement to the Journal of the Royal Statistical Society 7:1. https://doi.org/10.2307/2983630

38. Fieller EC (1954) Some problems in interval estimation. Journal of the Royal Statistical Society: Series B

(Methodological) 16:175–185. https://doi.org/10.1111/j.2517-6161.1954.tb00159.x

39. von Luxburg U, Franz VH (2004) Confidence Sets for Ratios: A Purely Geometric Approach To Fieller's Theorem. Max Planck Institute for Biological Cybernetics

40. Taneera J, Fadista J, Ahlqvist E, et al (2015) Identification of novel genes for glucose metabolism based upon expression pattern in human islets and effect on insulin secretion and glycemia. Hum Mol Genet 24:1945–1955. https://doi.org/10.1093/hmg/ddu610

41. Ottosson-Laakso E, Krus U, Storm P, et al (2017) Glucose-Induced Changes in Gene Expression in Human Pancreatic Islets: Causes or Consequences of Chronic Hyperglycemia. Diabetes 66:3013–3028. https://doi.org/10.2337/db17-0311

42. Hall E, Dekker Nitert M, Volkov P, et al (2018) The effects of high glucose exposure on global gene expression and DNA methylation in human pancreatic islets. Mol Cell Endocrinol 472:57–67. https://doi.org/10.1016/j.mce.2017.11.019

Supplemental Tables

Donor	HP18227	HP19208
Age (years)	36	35
Sex	Male	Male
Body mass index (kg/m^2)	27.6	21.7
HbA$_{1c}$ (mmol/mol)	40	36
HbA$_{1c}$ (%)	5.8%	5.4%
Source of islets	Prodo Labs	Prodo Labs
History of diabetes	No	No
Cause of death	Trauma (vehicle accident)	Stroke
Islet purity (%)	85%	85%
Islet viability (%)	95%	95%
10x Genomics reagent kit	SC3'v2	SC3'v3

ESM Table 1. Donor characteristics. Characteristics of the human pancreatic islet donors for this experiment.

	HOPX	ERO1B
gRNA#1	GGGCGAGATAGATGATTCCGCGG	CGGCCCAGGCGACGACCCAAGGG
gRNA#2	GTCCGCGACTAGCCGGCCTCCGG	TCCCGAGTCCTCTCGTTCAGCGG
gRNA#3	GACTTGACAGATCGCGAGGGTGG	CCAGCGGCCGAGCGACTCCAGGG

	RHOBTB3	HNRNPA2B1
gRNA#1	ATATTCCGCGGCGCCCCGCGCGG	TACGCGACGGCCTGACGTAGCGG
gRNA#2	ACAAAGGAGCCGCCCGCGCGGGG	TGAAGCGACTGAGTCCGCGATGG
gRNA#3	GAGTGCCCGGGAACATCGCCGGG	GTCCGGTTCGTGTTCGTCCGCGG

	Non-target
gRNA#1	GCGGGCAGAACGACCCTGAC
gRNA#2	GTAGGCGCGCCGCTCTCTAC

ESM Table 2. gRNA sequences. Guide RNA sequences for *HOPX*, *ERO1B*, *RHOBTB3*, *HNRNPA2B1*, and negative controls.

		Sequence (5'→ 3')
HOPX	Forward primer	ACAGAGGACCAGGTGGAAAT
	Reverse primer	GGGTCTCCTCCTCGGAAAG
ERO1B	Forward primer	GCTTATATCGGGACTTCATGCT
	Reverse primer	GGACCTTCTCCCTTGGTTTC
RHOBTB3	Forward primer	GAGTGTCCTGATTCCCGTTTAT
	Reverse primer	GTCTGGACACTTGGTACATCTC
HNRNPA2B1	Forward primer	GGCTTAAGCTTTGAAACCACAG
	Reverse primer	CAGCATCAACCTCAGCCAT
GAPDH	Forward primer	CTGGGCTACACTGAGCACC
	Reverse primer	AAGTGGTCGTTGAGGGCAATG

ESM Table 3. qRT-PCR primers. Sequences used as forward and reverse primers for qRT-PCR experiments targeting *HOPX*, *ERO1B*, *RHOBTB3*, *HNRNPA2B1*, and *GAPDH*.

ESM Table 4. Differentially expressed immediate early genes (IEGs) across cell types and models. IEGs from PMID:28960196. See ESM Tables excel sheet.

ESM Table 5. Differentially expressed glucose-associated genes. Overlap of glucose-associated genes from this study across cell types (any glucose-related model) and other bulk RNA-seq islet studies. See ESM Tables excel sheet.

Supplemental Figures

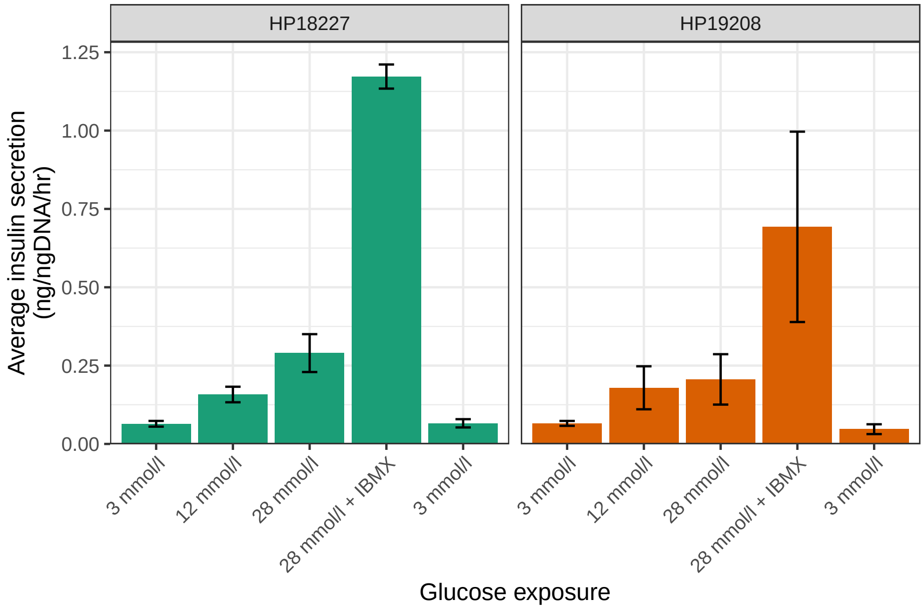

ESM Fig. 1. Insulin secretion after glucose exposure. Average insulin secretion (y-axis) after sequential exposure to different glucose concentrations (x-axis) for both donors (facet; Methods). Error bars denote 95% confidence intervals.

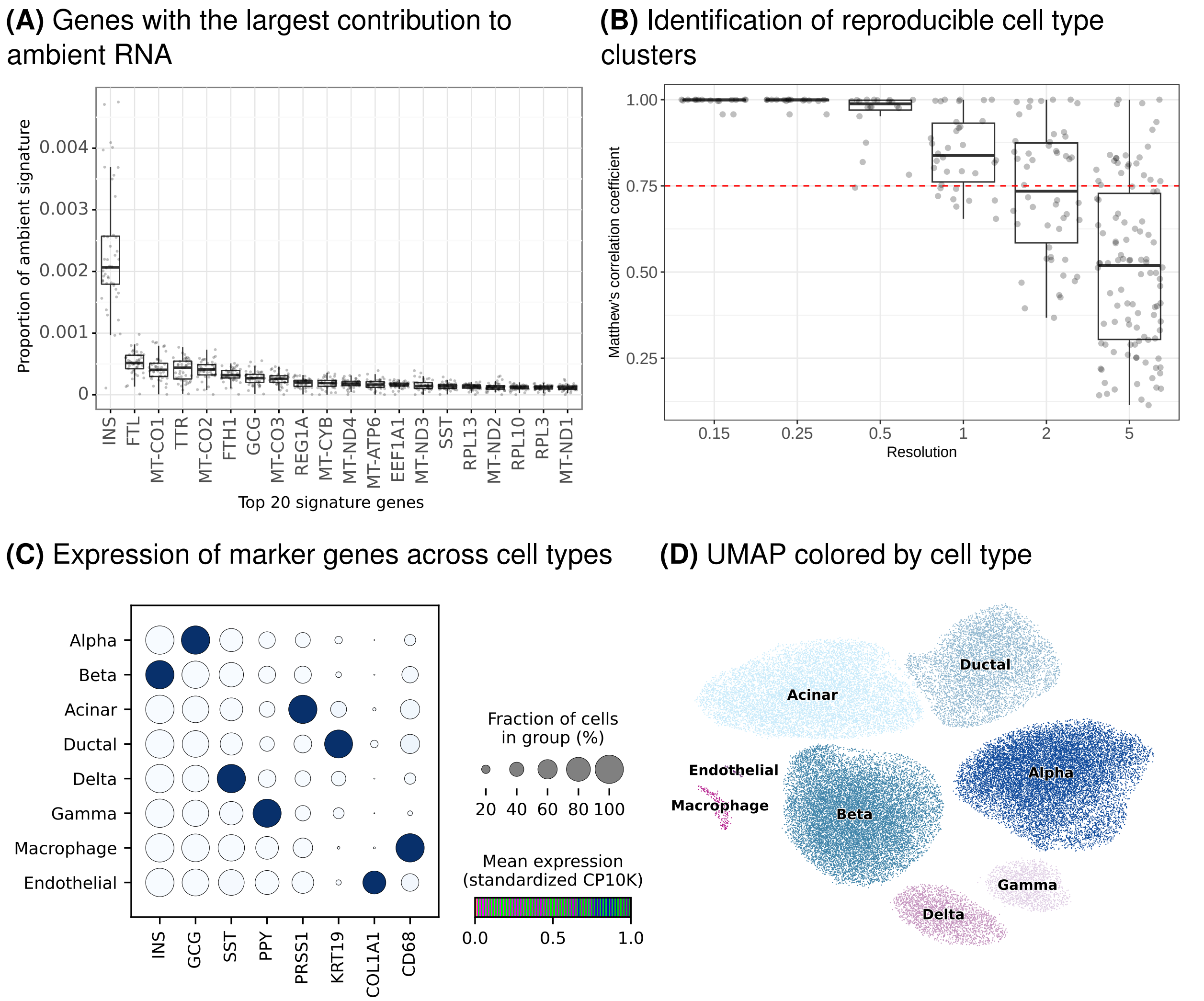

ESM Fig. 2. Summary of single-cell RNA-seq data and cell type clusters. (A) 20 genes (x-axis) with the largest contribution to the ambient RNA signature (y-axis) across samples. (B) Distribution of cluster (points) predictability measured using Matthew's correlation coefficient (y-axis) across different clustering resolution values (x-axis). Dashed line at 0.75. (C) Expression of cell type marker genes (x-axis) across final clusters (y-axis). (D) Uniform manifold approximation and projection (UMAP) dimensions colored by cell type.

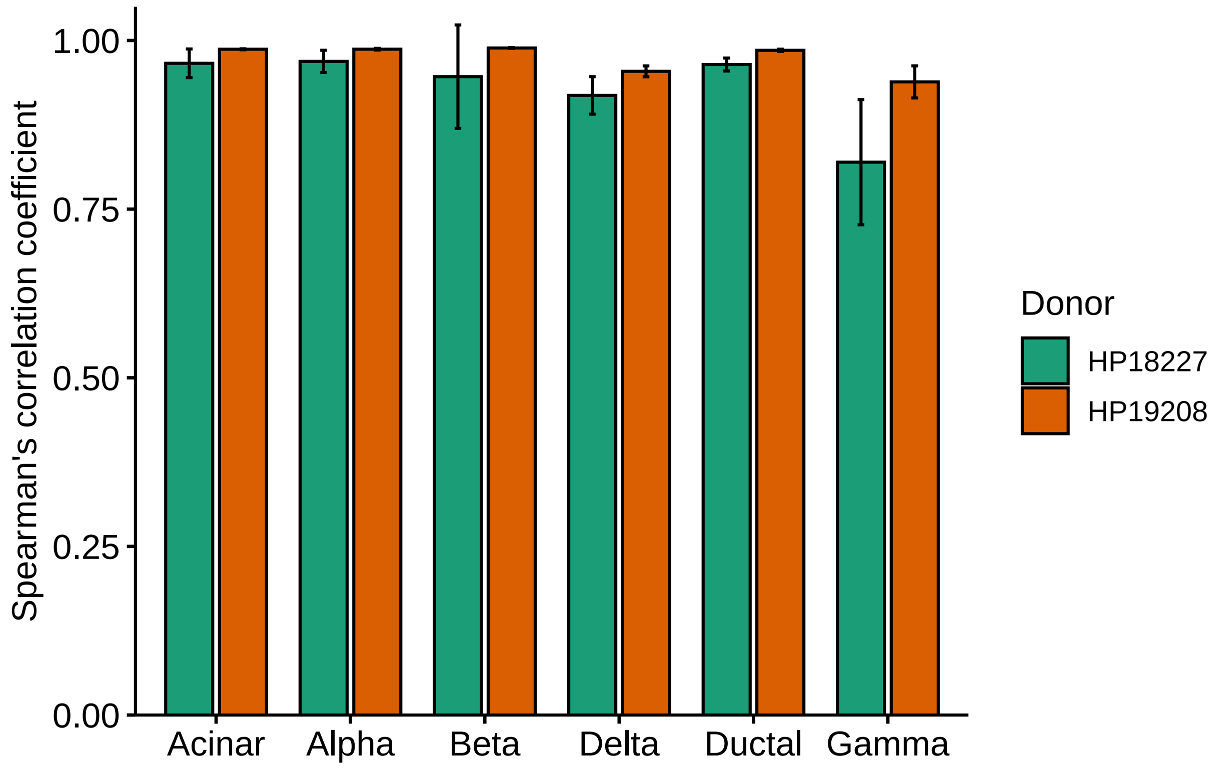

ESM Fig. 3. Gene expression correlation between replicates. Average correlation (y-axis) of gene expression between replicates of each donor (color) for each cell type (x-axis) at each time point and glucose condition. Error bars denote 95% confidence intervals.

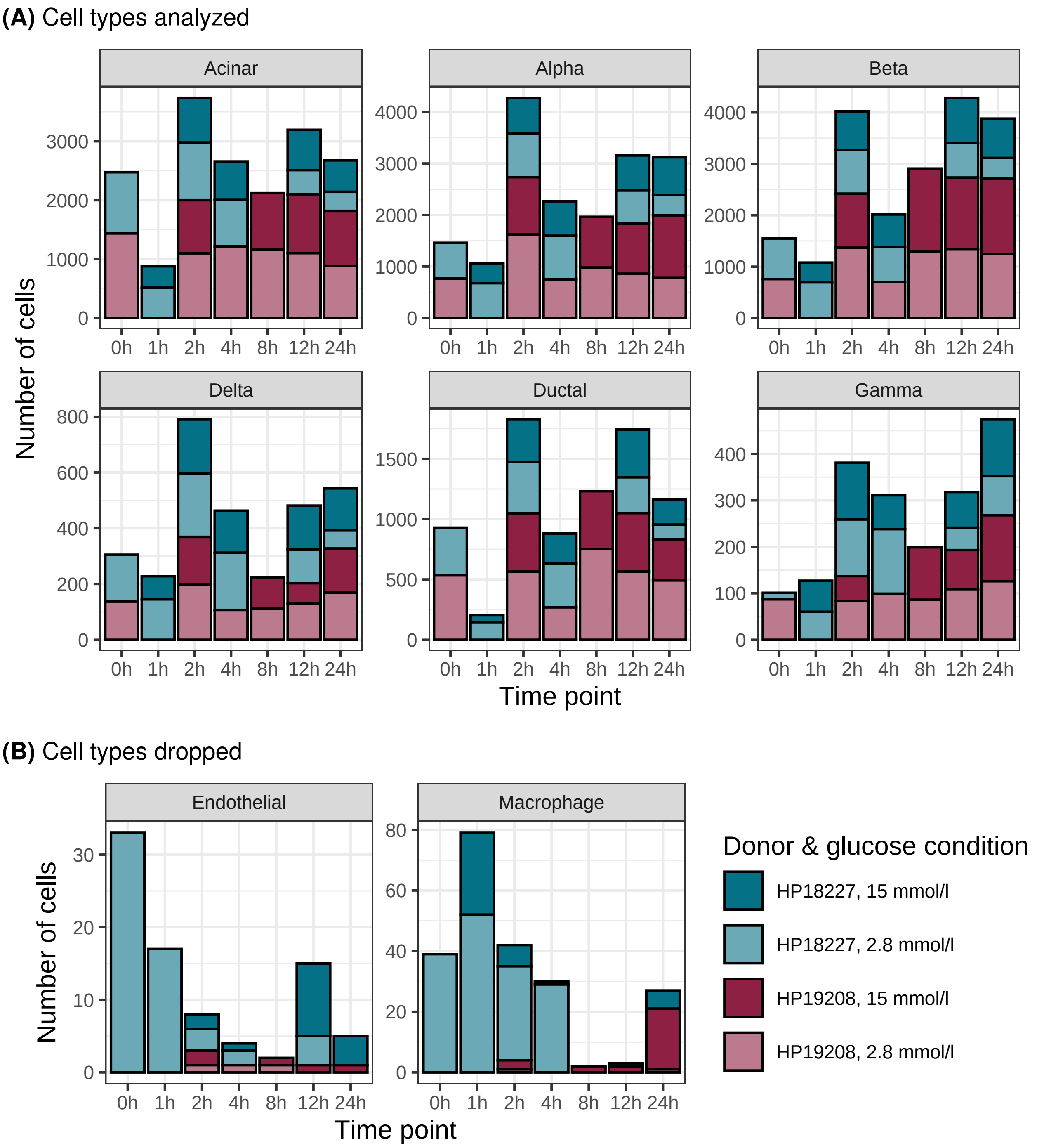

ESM Fig. 4. Donor representation within cell types. Number of cells (y-axis) from each donor (colors) across cell types (facets) at each time point (x-axis). Darker shades represent donor cells from the higher glucose exposure, and lighter shades represent lower glucose exposure. (A) Cell types considered in analyses. (B) Low-frequency cell types dropped from analyses.

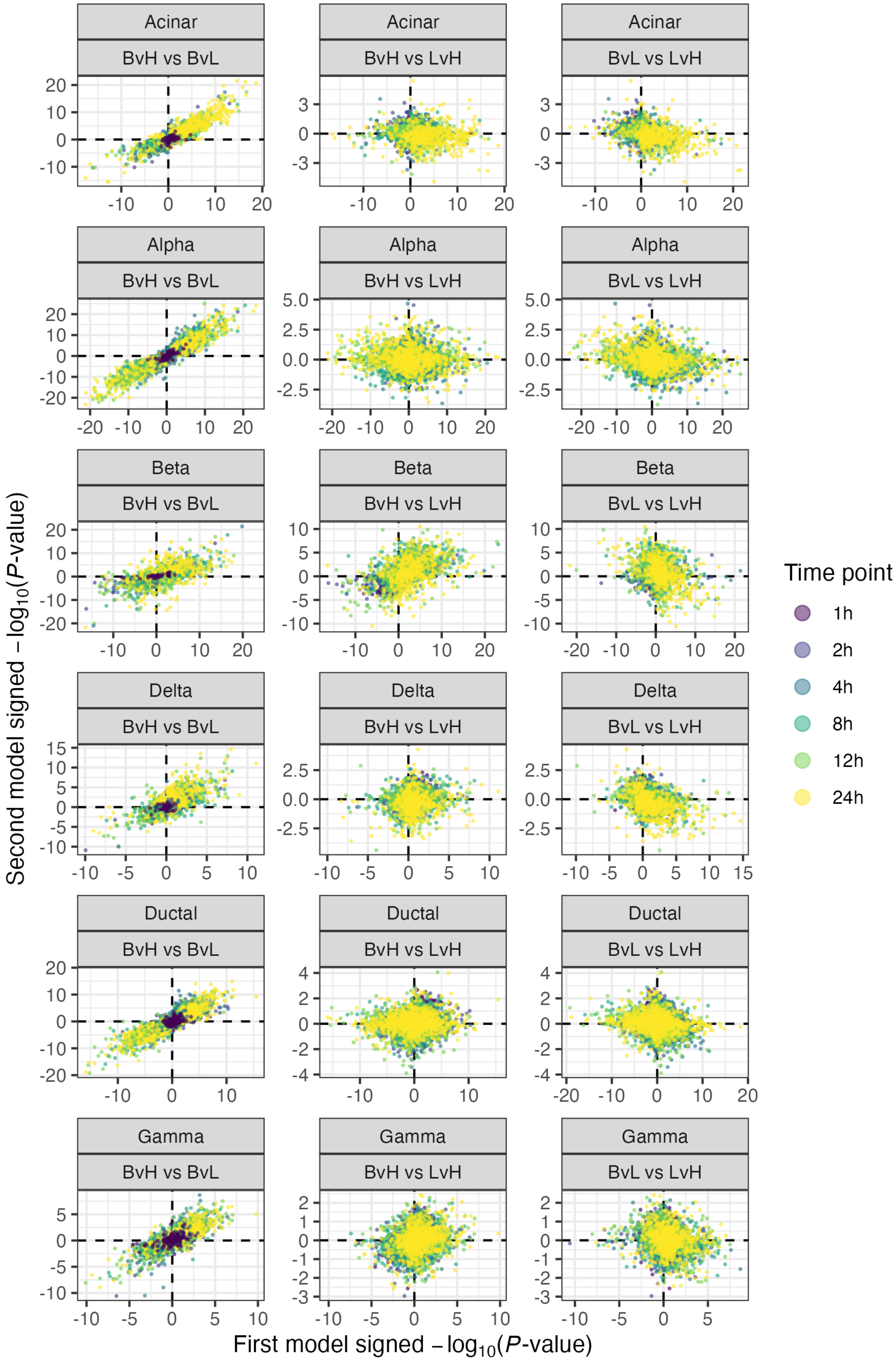

ESM Fig. 5. Comparison of differential gene expression results across discrete time models. Signed -$\log_{10}$(*P*-values) of BvL, BvH, and LvH models for each cell type (facets). First model in facet corresponds to the x-axis, and second model in facet corresponds to the y-axis.

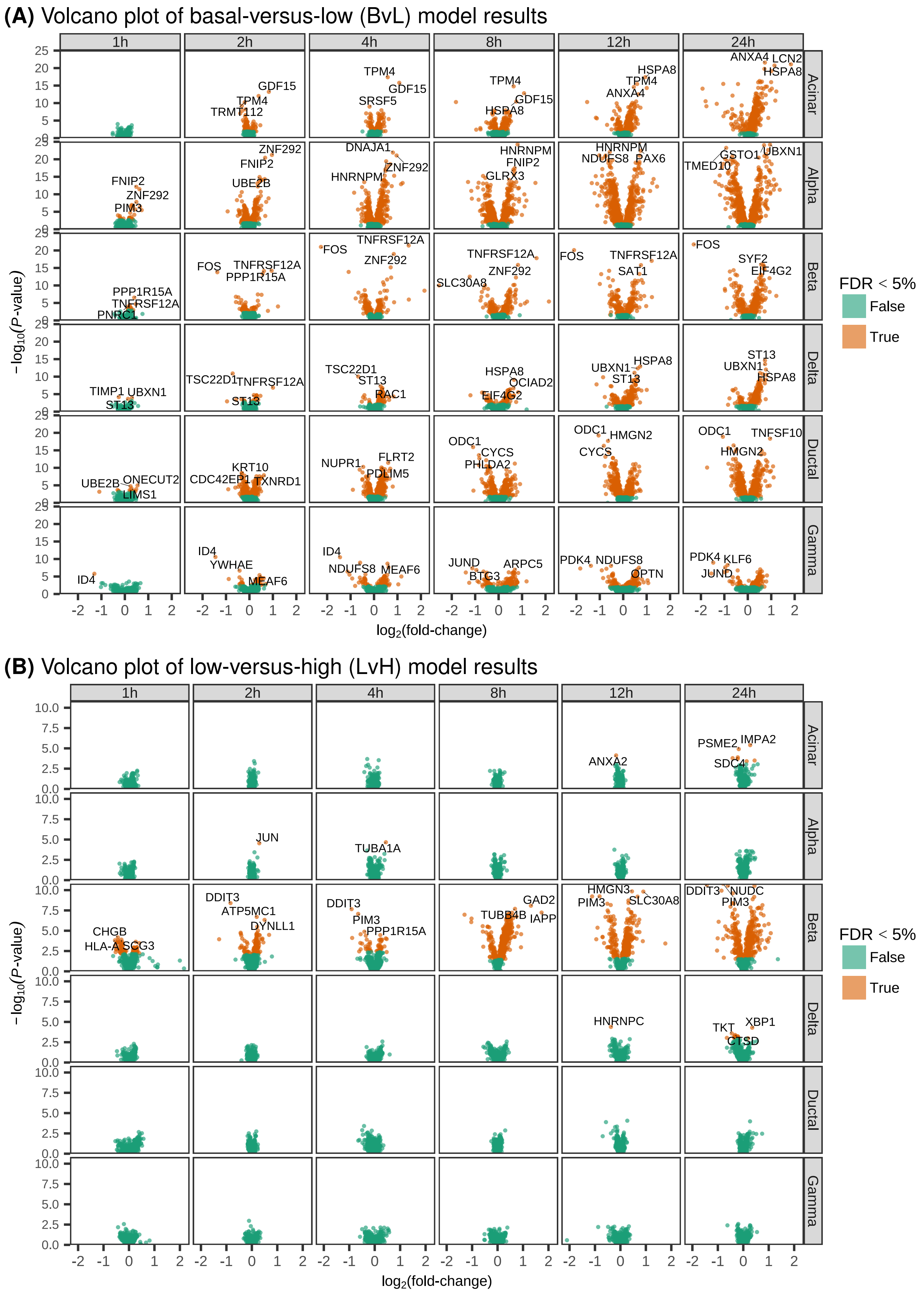

ESM Fig. 6. Volcano plot of BvL and LvH model results. -$\log_{10}$(*P*-values) (y-axis) and $\log_2$(fold-change) (x-axis) for all genes analyzed in the differential expression analyses across time points and cell types (facets). Top 3 differentially expressed genes (FDR<5%) with smallest *P*-values labelled. (A) BvL model. (B) LvH model.

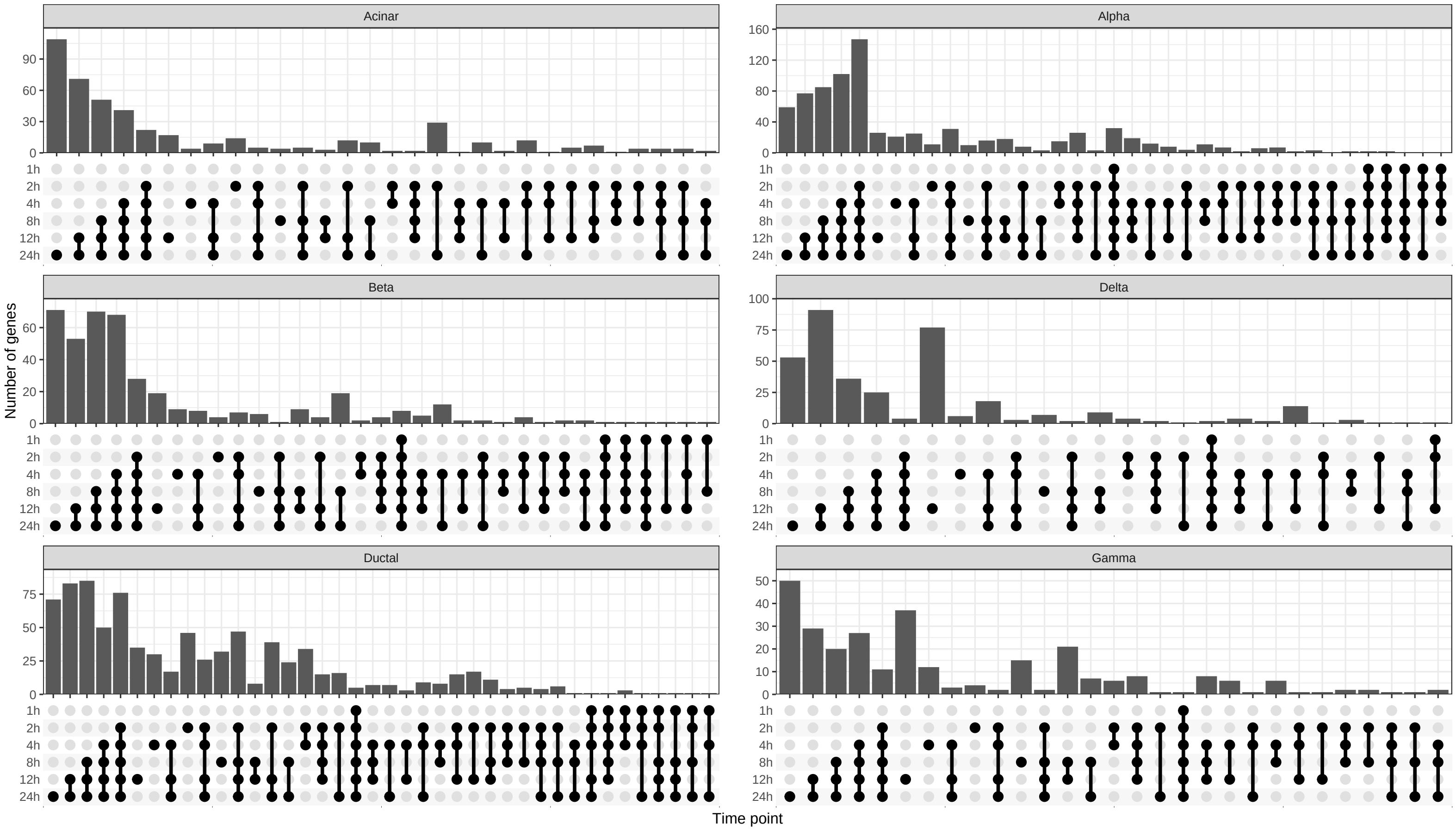

ESM Fig. 7. Overlap of differentially expressed genes in BvL models across time points. Number of associated genes (FDR<5%; y-axis) shared between time points (x-axis) in the BvL model within each cell type (facets).

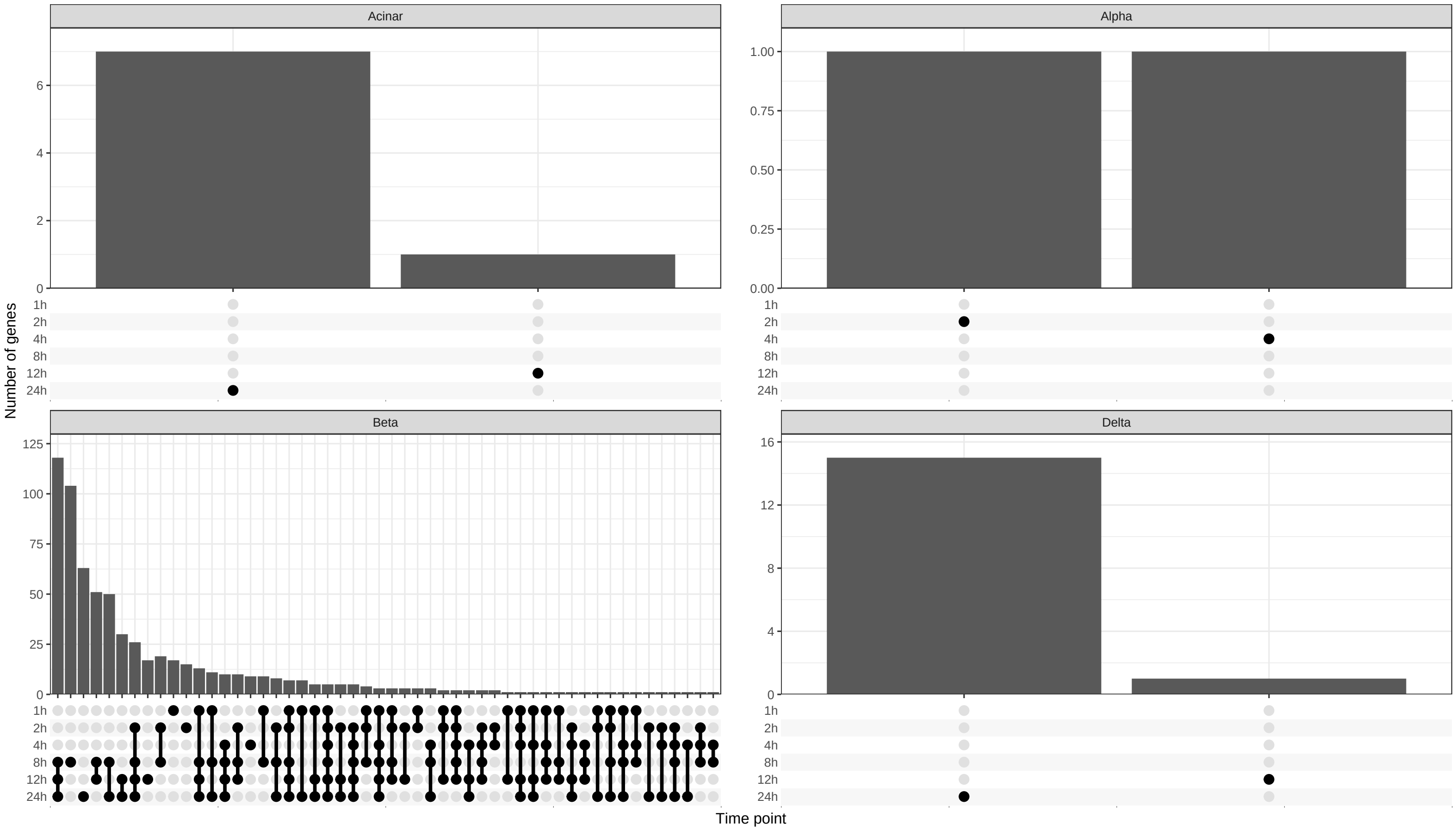

ESM Fig. 8. Overlap of differentially expressed genes in LvH models across time points. Number of associated genes (FDR<5%; y-axis) shared between time points (x-axis) in the LvH model within each cell type (facets).

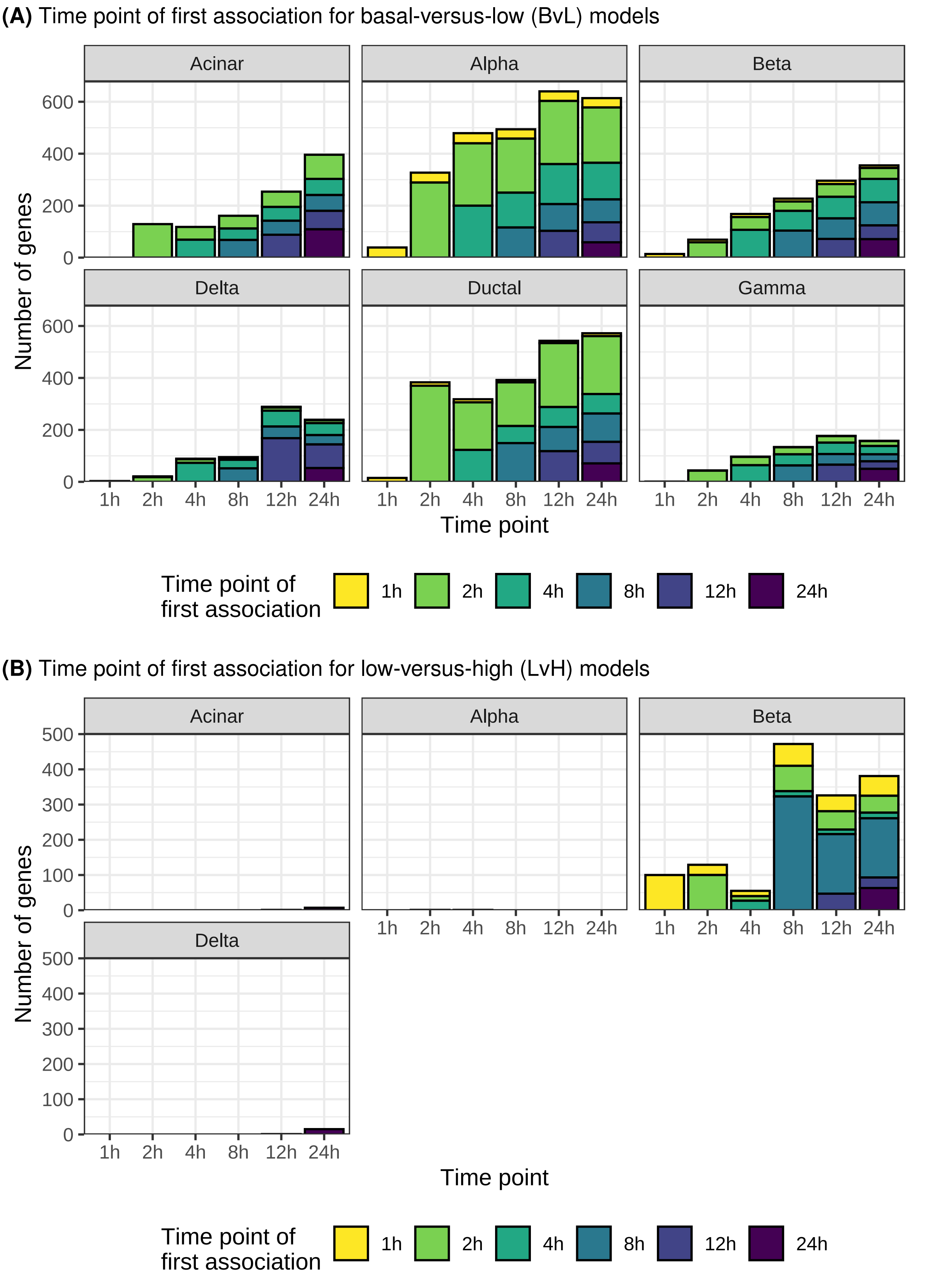

ESM Fig. 9. Time point of first association for BvL and LvH models. Number of associated genes (FDR<5%; y-axis) for each time point (x-axis) across cell types (facets). Color denotes the time point where the gene was first identified as differentially expressed. (A) BvL model. (B) LvH model.

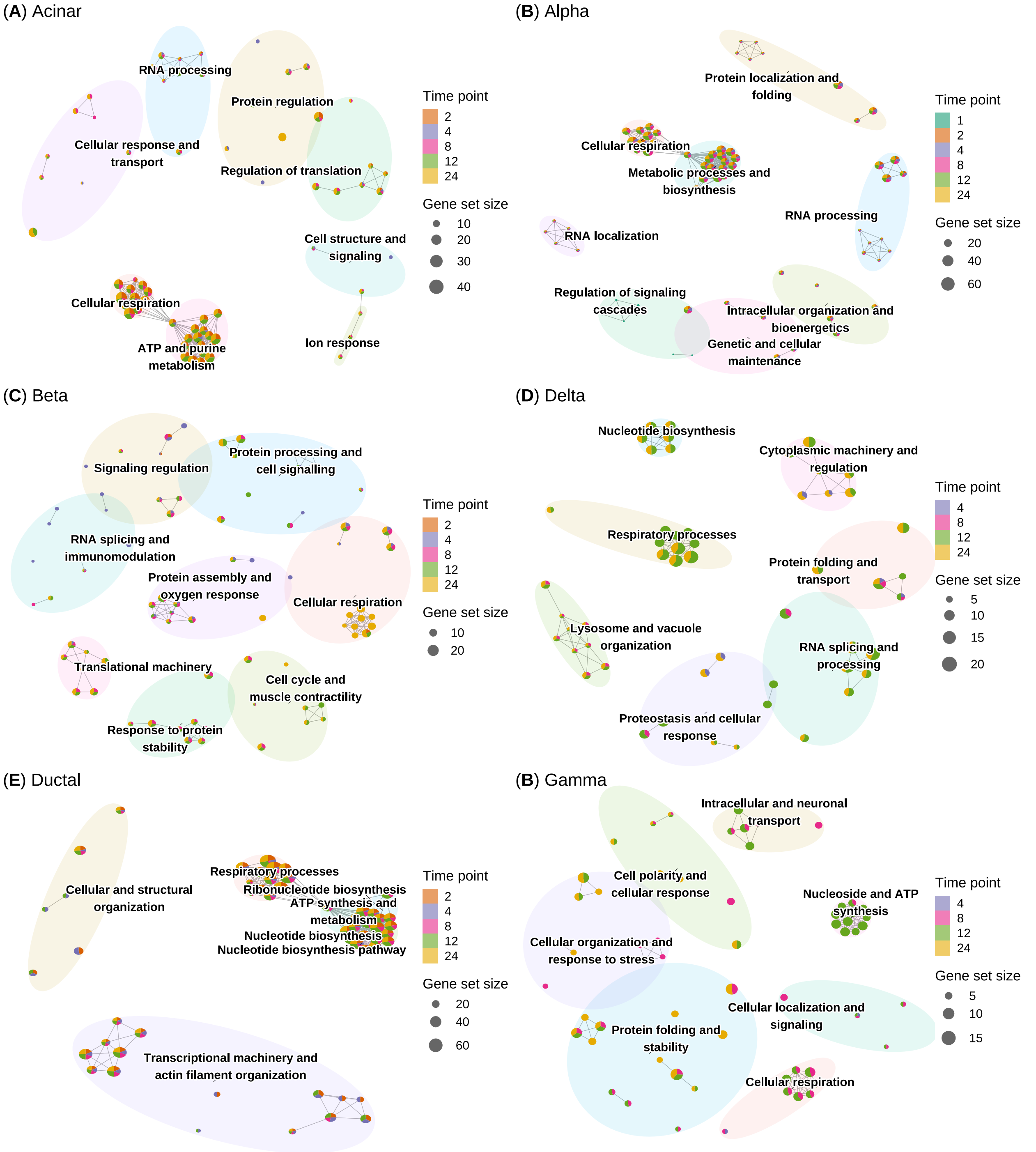

ESM Fig. 10. Network of GO terms enriched in BvL models. Network of GO terms enriched (FDR<5%) in differential expression results from BvL models across cell types. Nodes represent GO terms. Pie charts represent the proportion of time point-associated genes within each GO term. Node size represents the number of genes overlapping each GO term. Edges represent the similarity between nodes, with thickness and shorter lengths denoting stronger similarity. Clusters defined using similarity between nodes (Methods).

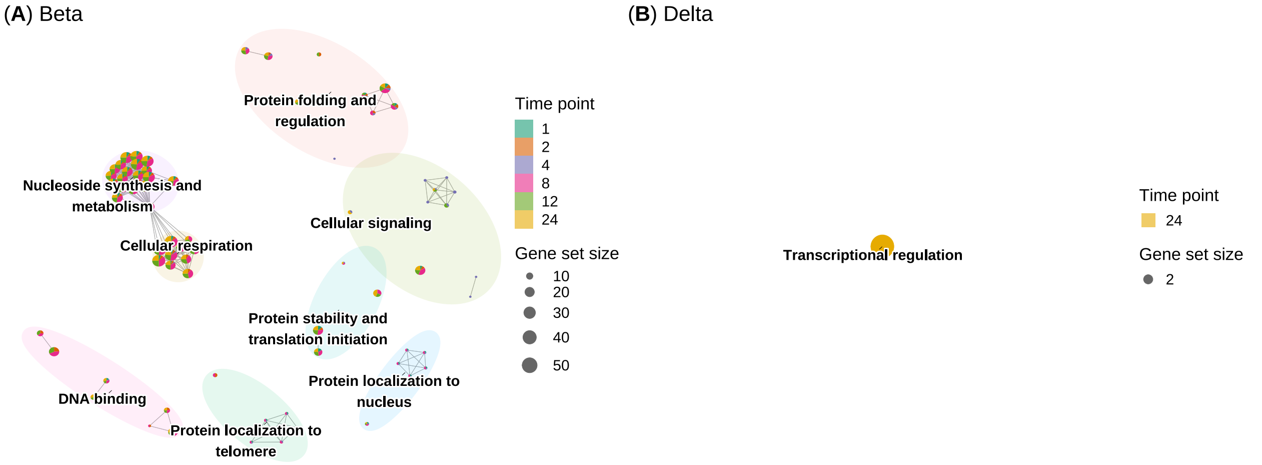

ESM Fig. 11. Network of GO terms enriched in LvH models. Network of GO terms enriched (FDR<5%) in differential expression results from LvH models across cell types. Nodes represent GO terms. Pie charts represent the proportion of time point-associated genes within each GO term. Node size represents the number of genes overlapping each GO term. Edges represent the similarity between nodes, with thickness and shorter lengths denoting stronger similarity. Clusters defined using similarity between nodes (Methods).

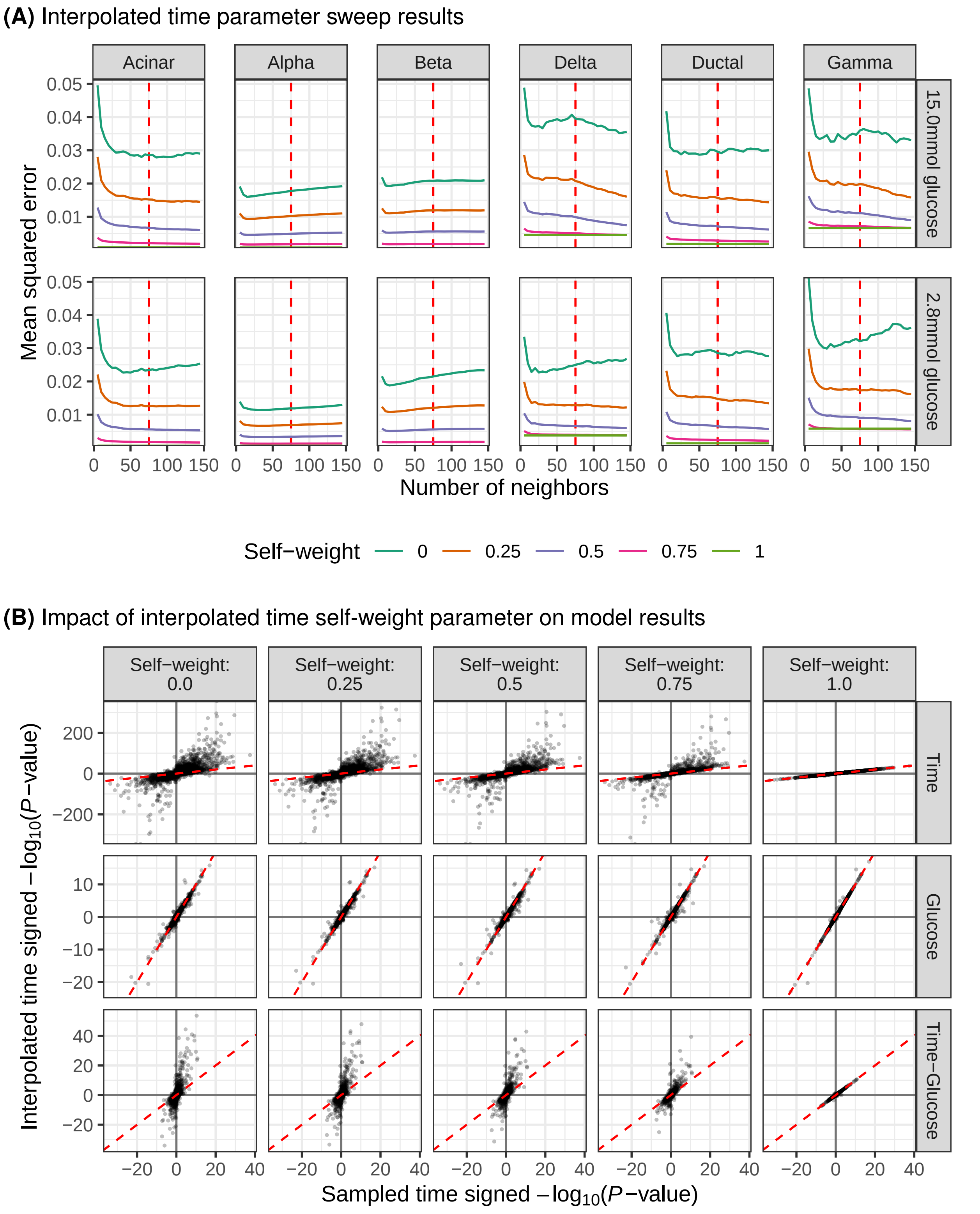

ESM Fig. 12. Evaluation of parameters used to calculate interpolated time. (A) Mean squared error (y-axis) calculated by comparing the per-cell interpolated time for each "number of neighbors" value (x-axis) to the per-cell interpolated time of the previous number of neighbors. Line colors correspond to different self-weight values. Red dashed line at 75. (B) Comparison of signed -$\log_{10}$(*P*-values) across continuous models (row facets) when using sampled time (x-axis) and interpolated time (y-axis) across self-weight values (column facets). Number of neighbors set to 75 for interpolated time calculation. Red line corresponds to the identity line.

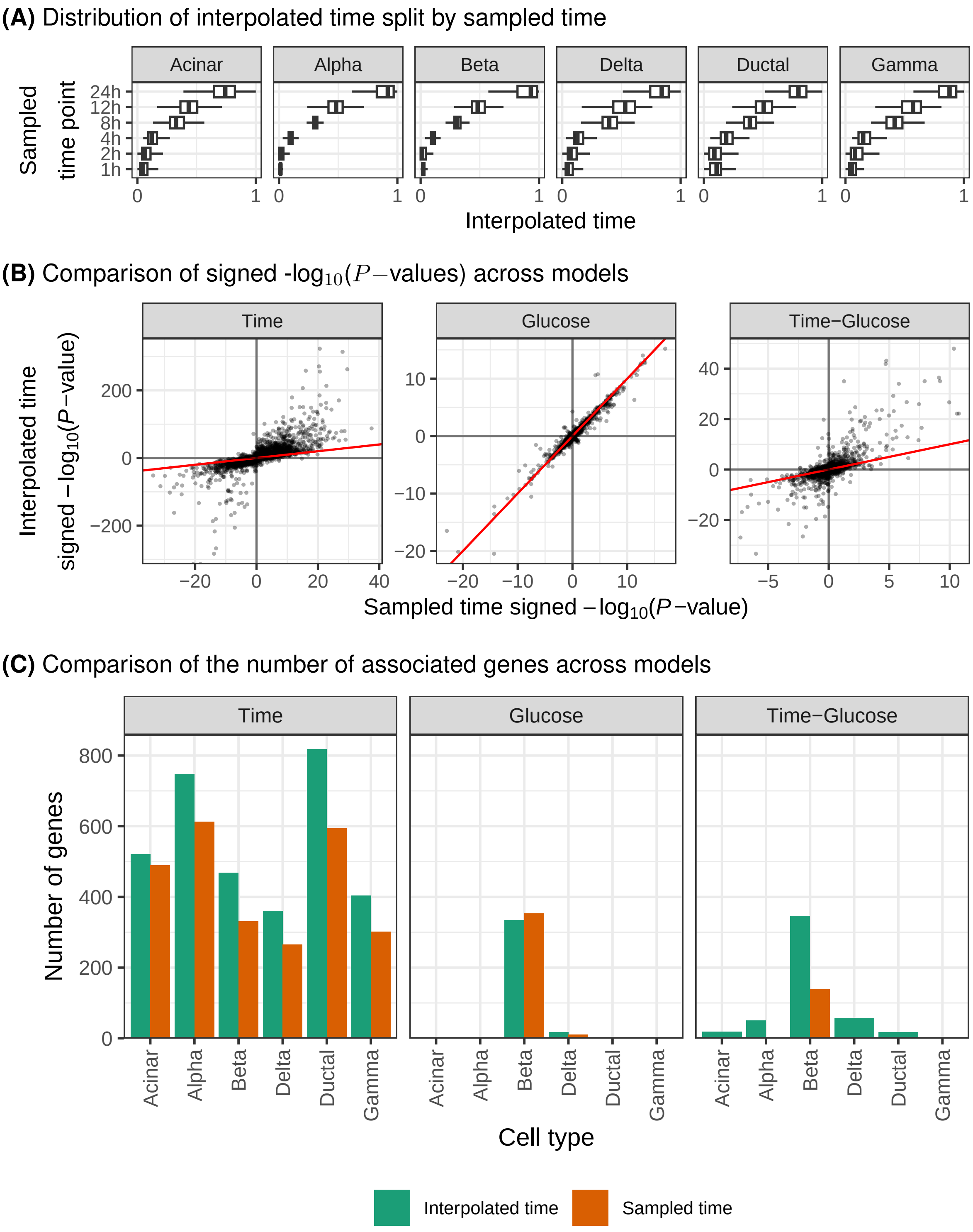

ESM Fig. 13. Comparison of differential gene expression results for continuous models using interpolated or sampled time. (A) Distribution of interpolated time (x-axis) for cells from each sampled time point (y-axis) across cell types. (B) Comparison of signed -$\log_{10}$(P-values) of differential gene expression results for continuous models using sampled time (x-axis) and interpolated time (y-axis). Red line corresponds to the identity line. (C) Number of associated genes (FDR<5%; y-axis) for each cell type (x-axis) across continuous models (facets). Color indicates if the model used interpolated or sampled time.

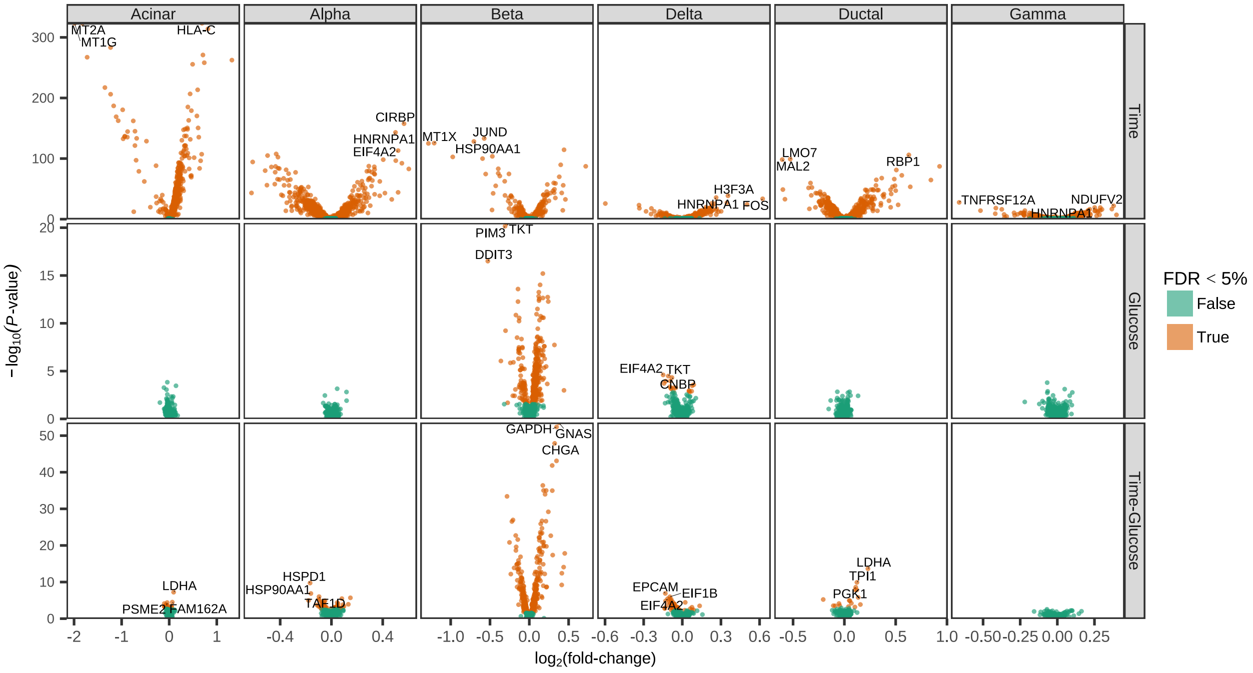

ESM Fig. 14. Volcano plot of results from continuous models. -log$_{10}$(*P*-values) (y-axis) and log$_2$(fold-change) (x-axis) for all genes analyzed in the differential expression analyses across cell types and models (facets). Top 3 differentially expressed genes (FDR<5%) with smallest *P*-values labelled.

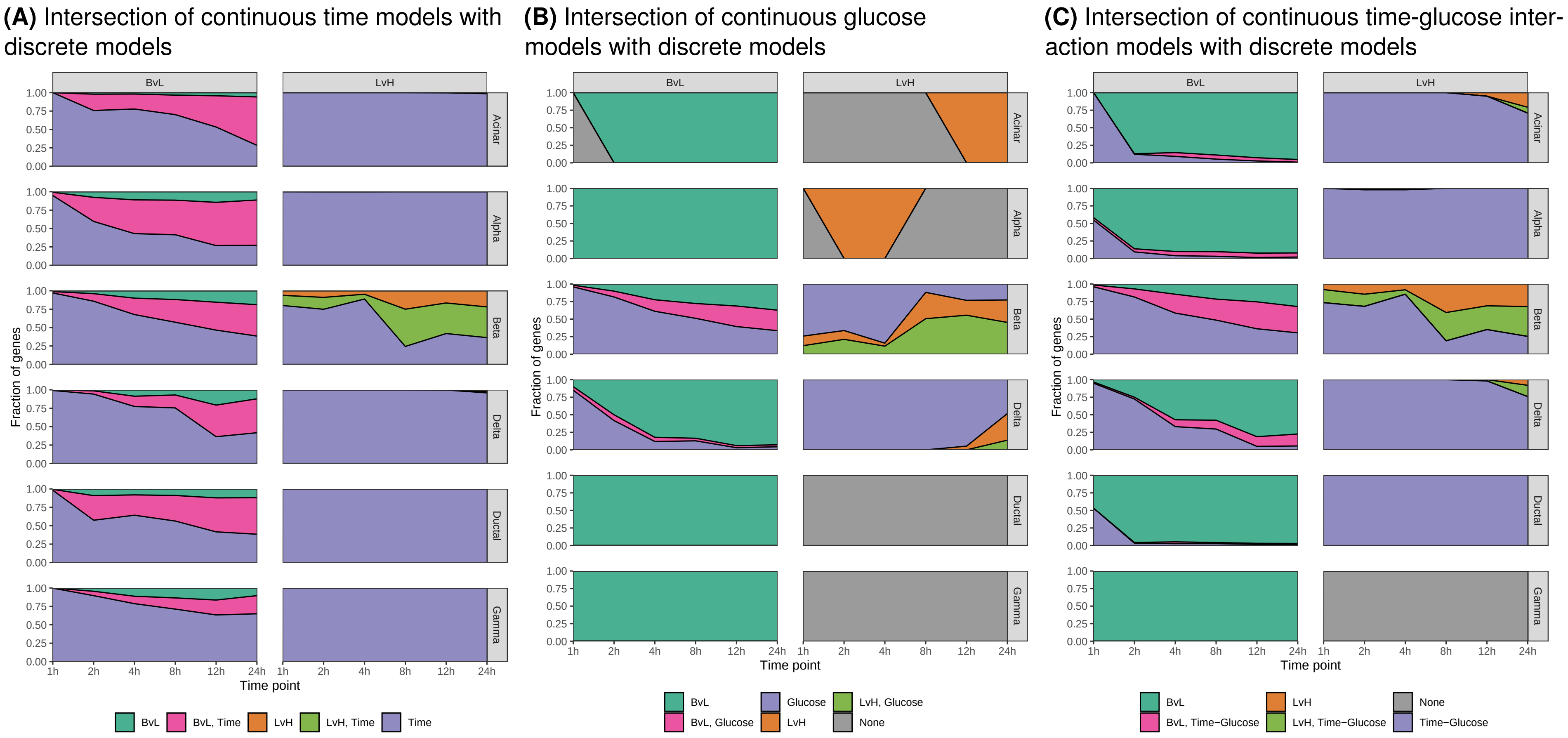

ESM Fig. 15. Intersection of differential gene expression results from continuous models with discrete models. Fraction of associated genes (FDR<5%; y-axis) shared between continuous models and discrete models (column facets) across cell types (row facets) and discrete model time points (x-axis). Color denotes the combination of models that genes belong to. (A) Continuous time model ("Time" in color legend). (B) Continuous glucose models ("Glucose" in color legend). (C) Continuous time-glucose interaction model ("Time-Glucose" in color legend).

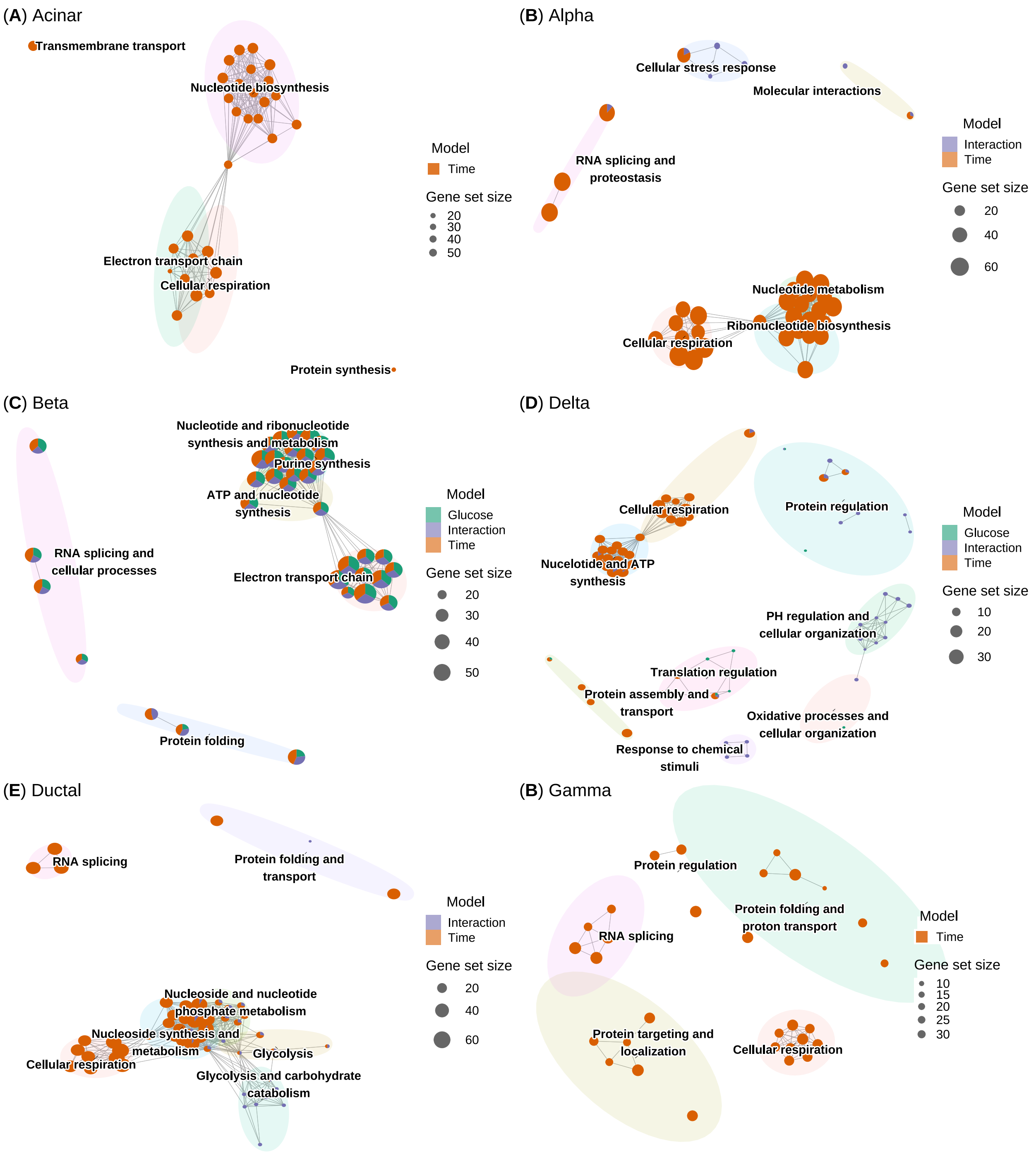

ESM Fig. 16. Network of GO terms enriched in continuous models. Network of GO terms enriched (FDR<5%) in differential expression results from continuous models across cell types. Nodes represent GO terms. Pie charts represent the proportion of model-associated genes within each GO term. Node size represents the number of genes overlapping each GO term. Edges represent the similarity between nodes, with thickness and shorter lengths denoting stronger similarity. Clusters defined using similarity between nodes (Methods).

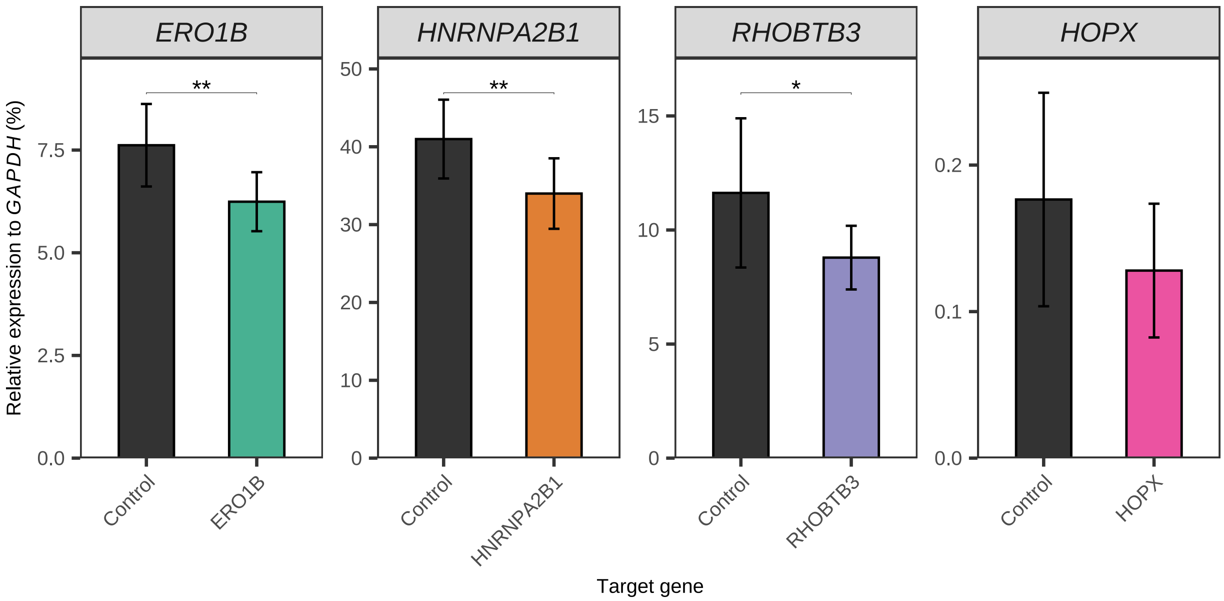

ESM Fig. 17. Transcriptional inhibition of target genes in EndoC-βH1. Relative expression of target genes to *GAPDH* (y-axis) after transcriptional inhibition (x-axis). * and ** denote $P<0.05$ and $P<0.01$, respectively (Welch's *t*-test). Error bars denote 95% confidence intervals.

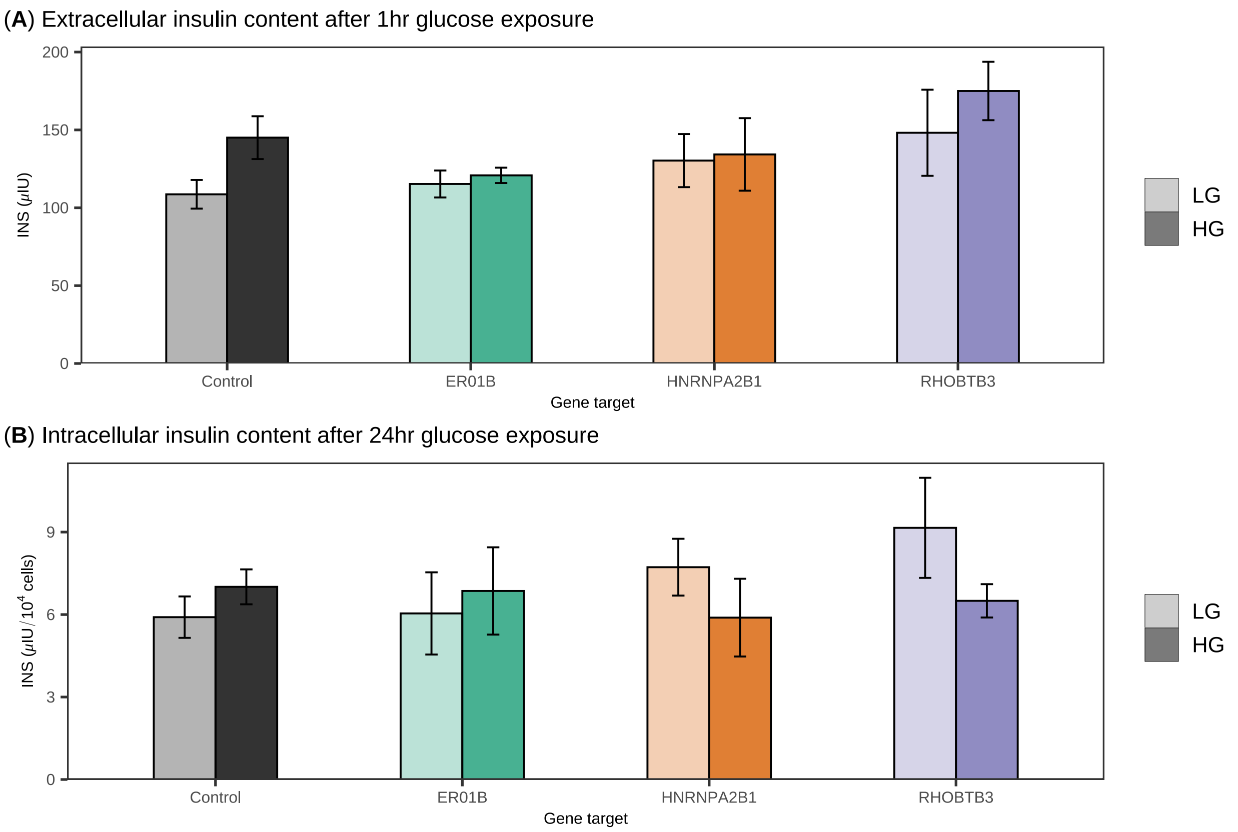

ESM Fig. 18. Insulin content after glucose exposure in control and CRISPRi EndoC-βH1 cells. (A) 1-hour extracellular insulin content (y-axis) and (B) 24-hour intracellular insulin content (y-axis) after low and high glucose exposure (shade) across control and CRISPRi EndoC-βH1 experiments (x-axis). Error bars represent 95% confidence intervals.

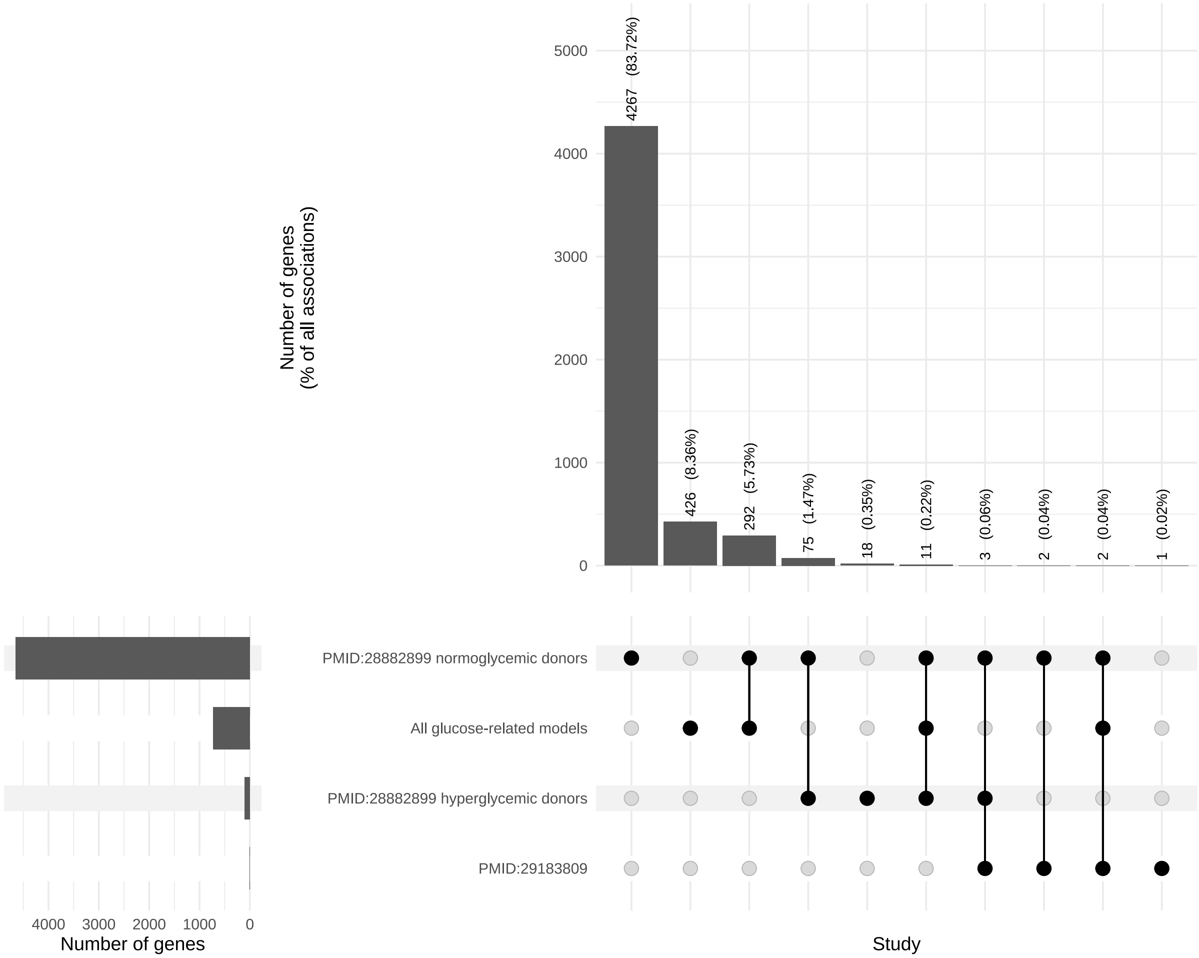

ESM Fig. 19. Overlap of differential gene expression results across studies. Number of associated genes (FDR<5%; y-axis) shared between studies (x-axis). Percent of all associations reported within parenthesis of main barplot. "All glucose-related models" refers to all glucose-related models reported in this study across all cell types and time points: LvH, continuous glucose, and continuous time-glucose interaction models.

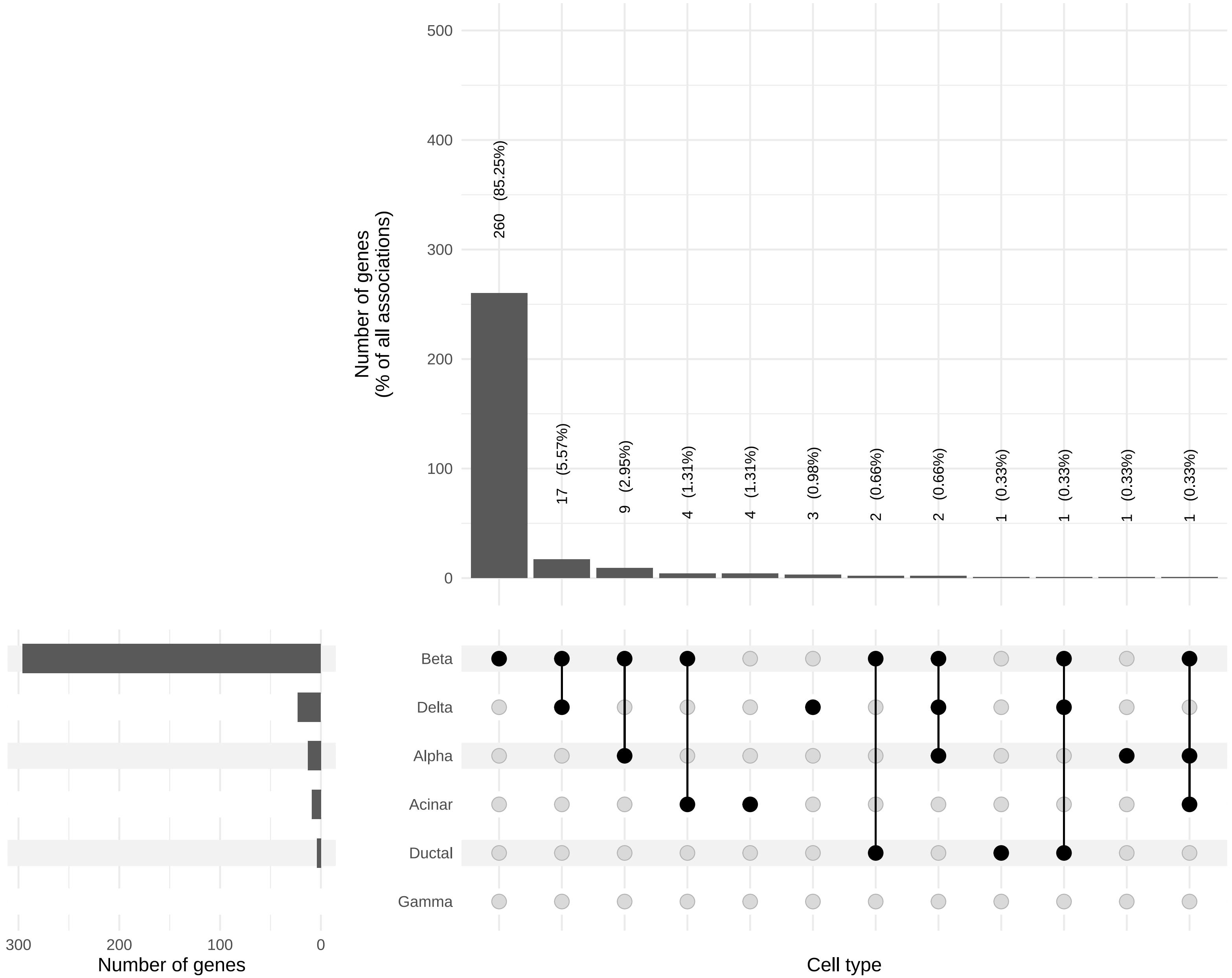

ESM Fig. 20. Overlap of differential gene expression results with normoglycemic donor results from PMID:28882899. Number of associated genes (FDR<5%; y-axis) from normoglycemic donors in PMID:28882899 identified in glucose-related models from this study (LvH, continuous glucose, and continuous time-glucose interaction models) across cell types (x-axis).

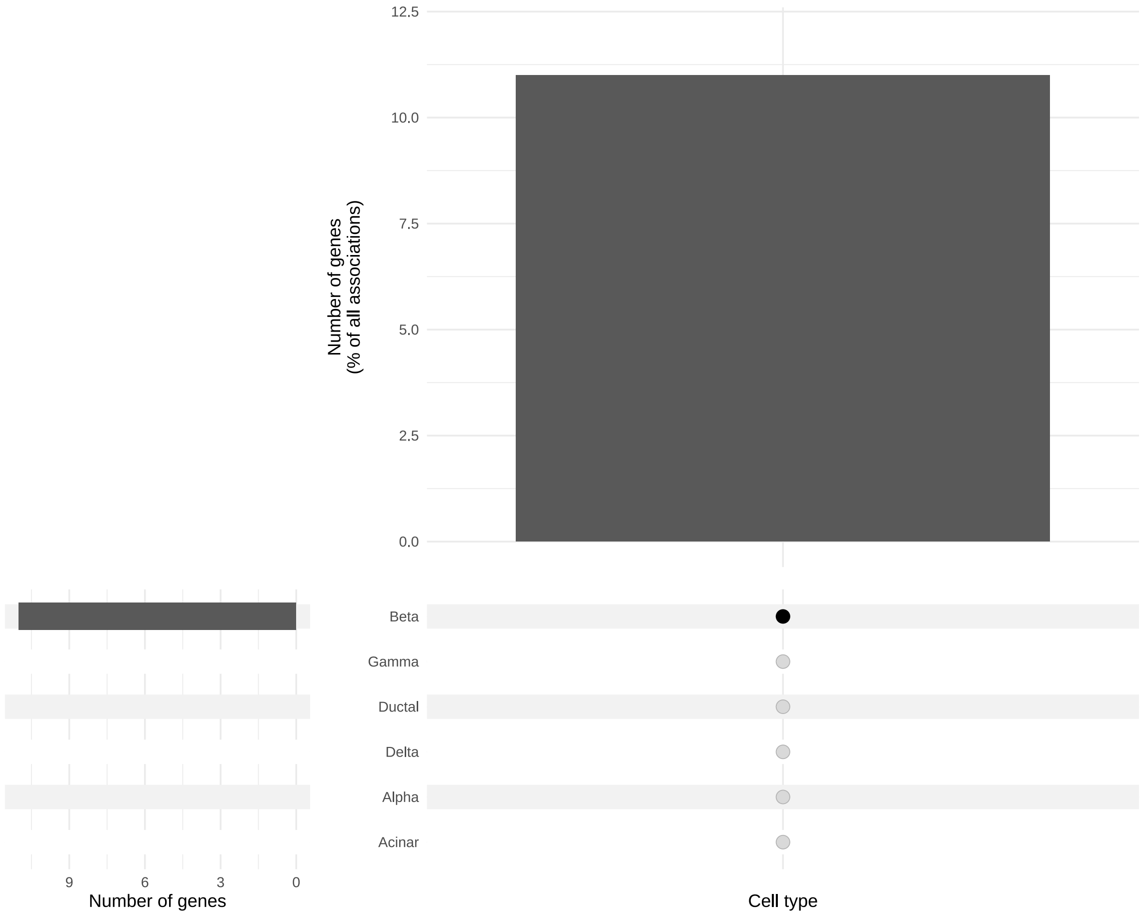

ESM Fig. 21. Overlap of differential gene expression results with hyperglycemic donor results from PMID:28882899. Number of associated genes (FDR$<$5%; y-axis) from hyperglycemic donors in PMID:28882899 identified in glucose-related models from this study (LvH, continuous glucose, and continuous time-glucose interaction models) across cell types (x-axis).

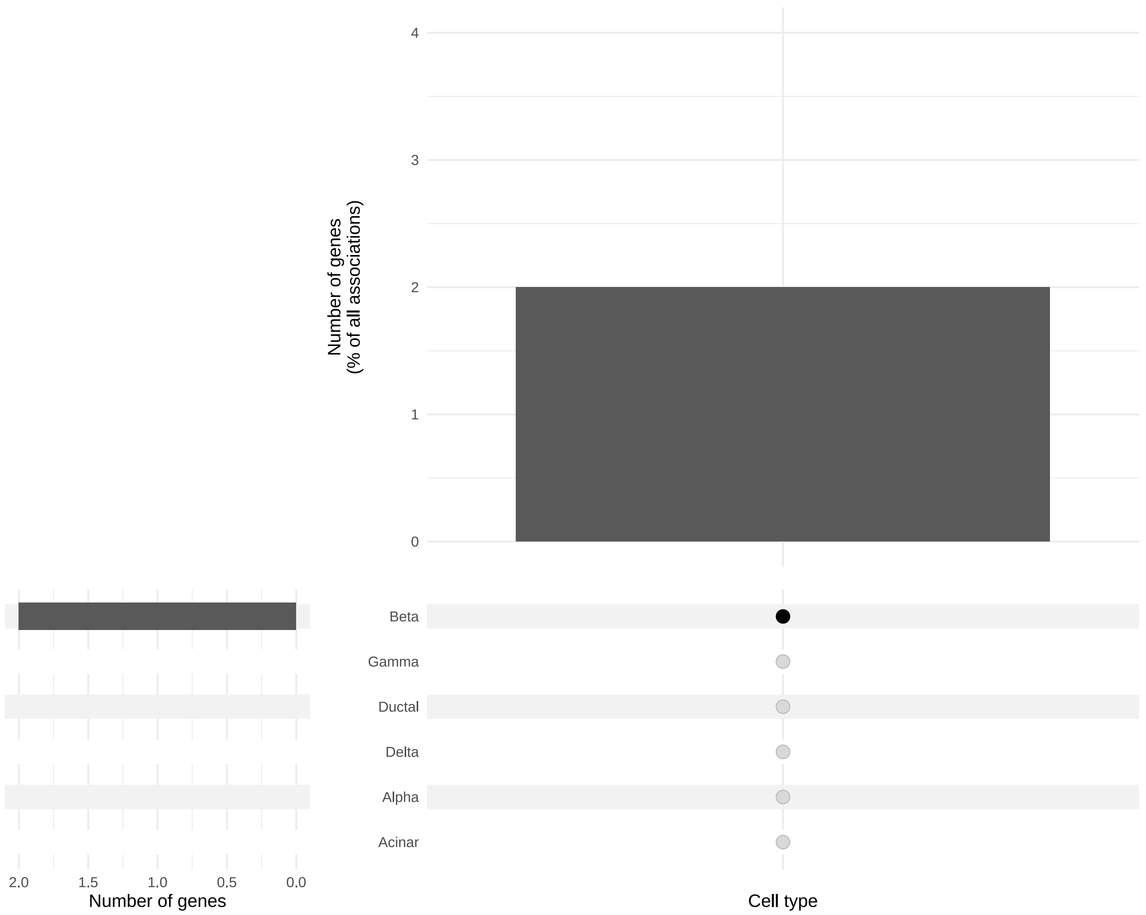

ESM Fig. 22. Overlap of differential gene expression results with results from PMID:29183809. Number of associated genes (FDR<5%; y-axis) in PMID:29183809 identified in glucose-related models from this study (LvH, continuous glucose, and continuous time-glucose interaction models) across cell types (x-axis).

Checklist for reporting human islet preparations used in research

Adapted from Hart NJ, Powers AC (2018) Progress, challenges, and suggestions for using human islets to understand islet biology and human diabetes. Diabetologia https://doi.org/10.1007/s00125-018-4772-2

**Islet preparation**	**1**	**2**	**3**	**4**	**5**	**6**	**7**	**8[a]**
**MANDATORY INFORMATION**								
Unique identifier	HP18227	HP19208						
Donor age (years)	36	35						
Donor sex (M/F)	Male	Male						
Donor BMI (kg/m^2)	27.6	21.7						
Donor HbA_{1c} or other measure of blood glucose control	40 mmol/mol	36 mmol/mol						
Origin/source of islets[b]	Prodo Labs	Prodo Labs						
Islet isolation centre								
Donor history of diabetes? Please select yes/no from drop down list	No	No						
**If Yes, complete the next two lines if this information is available**								
Diabetes duration (years)								
Glucose-lowering therapy at time of death[c]								
**RECOMMENDED INFORMATION**								
Donor cause of death	Trauma (vehicle accident)	Stroke						
Warm ischaemia time (h)								

Cold ischaemia time (h)								
Estimated purity (%)								
Estimated viability (%)								
Total culture time (h)[d]								
Glucose-stimulated insulin secretion or other functional measurement[e]								
Handpicked to purity? Please select yes/no from drop down list								
Additional notes								

[a]If you have used more than eight islet preparations, please complete additional forms as necessary
[b]For example, IIDP, ECIT, Alberta IsletCore
[c]Please specify the therapy/therapies
[d]Time of islet culture at the isolation centre, during shipment and at the receiving laboratory
[e]Please specify the test and the results
